# Changes in long-range rDNA-genomic interactions associate with altered RNA polymerase II gene programs during malignant transformation

**DOI:** 10.1038/s42003-019-0284-y

**Published:** 2019-01-28

**Authors:** Jeannine Diesch, Megan J. Bywater, Elaine Sanij, Donald P. Cameron, William Schierding, Natalie Brajanovski, Jinbae Son, Jirawas Sornkom, Nadine Hein, Maurits Evers, Richard B. Pearson, Grant A. McArthur, Austen R. D. Ganley, Justin M. O’Sullivan, Ross D. Hannan, Gretchen Poortinga

**Affiliations:** 1 Cancer Research Division, Peter MacCallum Cancer Centre, Melbourne, VIC 3000 Australia; 2 Department of Pathology, University of Melbourne, Parkville, VIC 3010 Australia; 3 ACRF Department of Cancer Biology and Therapeutics, John Curtin School of Medical Research, Australian National University, Canberra, ACT 2601 Australia; 4 Sir Peter MacCallum Department of Oncology, University of Melbourne, Parkville, VIC 3010 Australia; 5 Liggins Institute, The University of Auckland, Auckland, 1023 New Zealand; 6 Department of Biochemistry and Molecular Biology, Monash University, Clayton, 3800 VIC Australia; 7 Department of Biochemistry and Molecular Biology, University of Melbourne, Parkville, VIC 3010 Australia; 8 Department of Medicine, St Vincent’s Hospital, University of Melbourne, Fitzroy, VIC 3065 Australia; 9 School of Biological Sciences, The University of Auckland, Auckland, 1010 New Zealand; 10 School of Biomedical Sciences, University of Queensland, Brisbane, QLD 4072 Australia; 11 Present Address: Josep Carreras Leukaemia Research Institute, Barcelona, 08021 Spain; 12 Present Address: QIMR Berghofer Medical Research Institute, Brisbane, QLD 4029 Australia

## Abstract

The three-dimensional organization of the genome contributes to its maintenance and regulation. While chromosomal regions associate with nucleolar ribosomal RNA genes (rDNA), the biological significance of rDNA-genome interactions and whether they are dynamically regulated during disease remain unclear. rDNA chromatin exists in multiple inactive and active states and their transition is regulated by the RNA polymerase I transcription factor UBTF. Here, using a MYC-driven lymphoma model, we demonstrate that during malignant progression the rDNA chromatin converts to the open state, which is required for tumor cell survival. Moreover, this rDNA transition co-occurs with a reorganization of rDNA-genome contacts which correlate with gene expression changes at associated loci, impacting gene ontologies including B-cell differentiation, cell growth and metabolism. We propose that UBTF-mediated conversion to open rDNA chromatin during malignant transformation contributes to the regulation of specific gene pathways that regulate growth and differentiation through reformed long-range physical interactions with the rDNA.

## Abstract (web summary)

Jeannine Diesch et al. report the changes in rDNA chromatin state associated with cell transition into malignancy. They show that a specific transcription factor regulates this transition by altering rDNA chromatin, resulting in the reorganization of contacts between rDNA and the genome.

## Introduction

Advances in genomics and epigenomics have provided insights into three-dimensional (3D) genome organization at an unprecedented level of detail^1–3^, and highlight the dynamic relationship between genomic spatial organization and gene regulation^4,5^. However, relatively little is understood about how genome organization and gene expression are reshaped during disease development and their impact on the process^6^. The largest substructure in the nucleus is the nucleolus, the site of ribosome biogenesis, which forms dynamically around transcribed ribosomal RNA (rRNA) genes (rDNA) arranged in arrays of tandem repeats at chromosomal regions called nucleolar organizer regions (NORs)^7^. The NORs are organized on acrocentric chromosomes, with the highly variable rDNA copy number averaging more than 100 per diploid genome^7,8^. However, at any given time less than 50% of these rDNA copies are actively transcribed by the dedicated RNA polymerase I (Pol I) to produce the 47S rRNA precursor^9^. A large body of evidence supports a model of rDNA copies existing in one of three epigenetic chromatin states: silent (CpG methylated at the rDNA promoter), pseudo-silent (lacking hypomethylated promoters but transcriptionally inactive), or active (hypomethylated and transcriptionally competent)^10^. Active genes exhibit a range of transcription rates depending on the cellular state, and the relative ratio of silent, pseudo-silent and active genes is modulated during differentiation and development^11–15^. These states are regulated epigenetically and via the upstream binding transcription factor (UBTF), an architectural protein required to recruit Pol I to the rDNA promoter but also critical for binding to under-methylated, pseudo-silent rDNA repeats to convert them into the active, transcriptionally competent state^12,14,16,17^.

Functions beyond production of rRNA are well documented for the rDNA and nucleolus, including regulation of genomic stability and global gene expression^18–23^. Genomic sequences that include certain genes other than rDNA are localized to nucleoli in nucleolar-associated domains (NADs)^24–28^. The NAD nucleolar chromatin compartment is enriched for repressive chromatin marks and under-represented for active histone modifications, and NAD-associated genes are generally transcriptionally repressed^24–27^. This is consistent with evidence that the nucleolus is surrounded by a facultative heterochromatic shell^26,29,30^, and highlights a potential role for the nucleolus in dynamically regulating global gene transcription through nucleolar colocalization.

The interplay between altered nucleolar morphology and disease is well recognized and accelerated rRNA transcription and ribosome biogenesis is a common feature of many cancers^31–33^. This is reflected by the increased size and/or number of nucleoli in tumor cells, initially observed by pathologists over 100 years ago and used as a diagnostic and prognostic marker for certain cancers^34^. Moreover, the potential of dysregulated ribosome biogenesis as a therapeutic target in cancer has been demonstrated by the development of small molecule inhibitors of Pol I transcription^35–39^. The MYC oncogene, a potent transcriptional driver of growth-associated gene programs^40–42^ via all three RNA polymerases (Pol I, II, and III), has been implicated in sensitizing MYC-addicted cancer cells to inhibition of Pol I transcription^13,43–48^. However, while a substantial body of data has provided critical insight into our understanding of rDNA as a therapeutic target^36,37,39,49–53^, the precise mechanisms underlying the heightened sensitivity of tumor cells to perturbations in Pol I transcription and the degree to which subsequent disruption of nucleolar integrity contributes to the therapeutic window remain unresolved.

Here we examine whether changes to rDNA chromatin structure are associated with malignant transformation and further, are accompanied by alterations in rDNA-NAD interactions. Using the Eµ-*Myc* mouse model of spontaneous MYC-driven B-cell lymphoma, we showed that progression from premalignancy to malignancy in vivo is associated with UBTF-dependent epigenetic remodeling that activates a significant proportion of previously pseudo-silent rDNA repeats. Circularized chromosome conformation capture sequencing (4C-seq) demonstrated that, concomitant with activation of rDNA during malignancy, the population of genomic loci interacting with the rDNA changes during lymphomagenesis. Genes associated with these rDNA-interacting loci show an inverse relationship between their rDNA interaction level and their gene expression. A sub-population of the rDNA-NAD interactions that change during malignant progression require the active chromatin state of the rDNA repeats. Notably, of this NAD-associated loci sub-population, those with reduced expression in malignant cells are enriched for genes encoding proteins involved in B-cell differentiation. Conversely, those with increased expression are enriched for growth and metabolism gene ontologies. We propose that malignancy-associated alterations in the rDNA chromatin status are linked to formation of new rDNA-genomic interactions and co-occur with gene expression changes that contribute to the malignant phenotype.

## Results

### rDNA chromatin opens during Eµ-*Myc* malignant progression

**Fig. 1:**
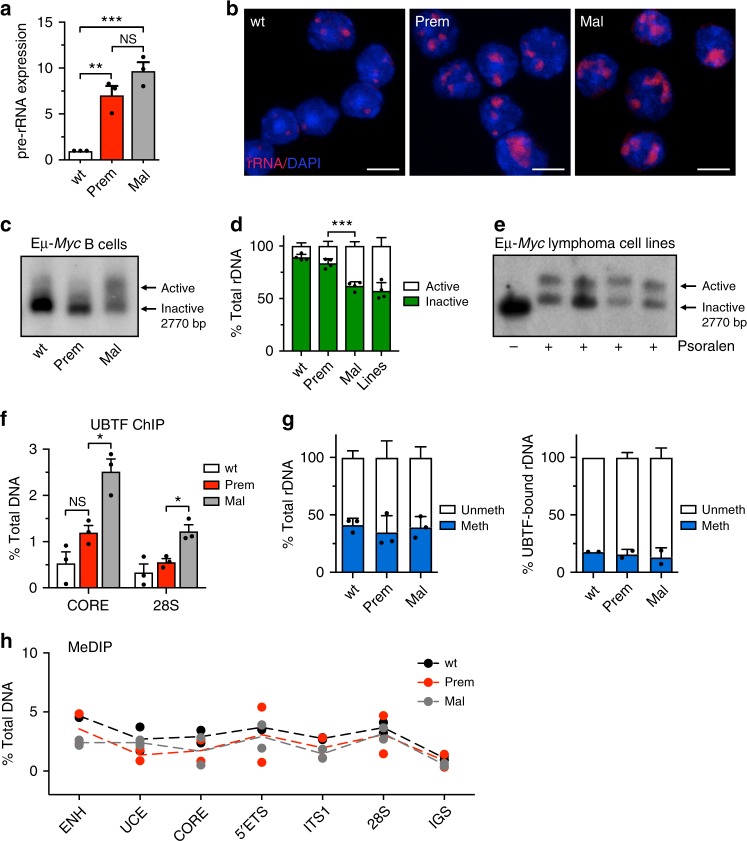
rDNA chromatin undergoes remodeling as Eμ-*Myc* cells transition to malignancy. **a** Relative pre-rRNA expression (rRNA transcription rate) in wild type (wt), premalignant (prem) and malignant (mal) Eμ-*Myc* cells determined by qRT-PCR using primers to the rapidly degraded 5’ external transcribed spacer (5’ETS) region. Pre-rRNA levels were normalized to *B2M* mRNA and represented relative to wt (${\bar{ x}}$ ± s.e.m., *n* = 3 mice/cell stage). **b** rRNA levels in cells from **a** as determined by rRNA FISH using a probe (red) to the degraded internal transcribed spacer (ITS1) region. Nuclei were counterstained with DAPI (blue). Scale bars, 10 μm. **c** Representative Southern blot of psoralen cross-linked genomic rDNA from wt, prem and mal Eμ-*Myc* cells; arrows indicate active and inactive rDNA repeats. **d** Four independent psoralen Southern blot experiments as in **c** were quantitated with ImageQuant (${\bar{ x}}$ ± s.e.m., *n* = 4). Also shown is quantitation (lines) of **e** Southern blot of psoralen cross-linked genomic rDNA from Eμ-*Myc* lymphoma cells lines (lane 1: no psoralen control, 4242 line; lanes 2–5: lines 226, 107, 4242, and 102); arrows indicate active and inactive rDNA repeats. **f** ChIP analysis of UBTF binding at the rDNA promoter (CORE) and transcribed (28S) regions. The percentage (%) of DNA immunoprecipitated with anti-UBTF or rabbit sera (RS) was calculated relative to the unprecipitated input control and the % of DNA associated with RS was subtracted from corresponding UBTF samples (${\bar{ x}}$ ± s.e.m., *n* = 3). **g** CpG methylation at the rDNA core promoter determined by HpaII digestion followed by qPCR of total rDNA (left panel) and UBTF-bound rDNA (right panel). The percentage of unmethylated and methylated rDNA is plotted (${\bar{ x}}$ ± s.d., total rDNA *n* = 3, UBTF ChIP-CHOP *n* = 2). **h** CpG methylation frequency across the rDNA unit determined by methylated DNA immunoprecipitation (MeDIP) followed by qPCR using amplicons spanning the rDNA repeat. All amplicon primers as labeled except: 5’ETS (ETS1), 28S (28S-2), and IGS (IGS2). Samples are analyzed by qPCR with results expressed as percent (%) of total DNA (minus RS % total) as described in **f** (${\bar{ x}}$ ± s.d., *n* = 2). The Student's *t*-test was used for all statistical analyses: *p-value < 0.05; **p-value < 0.01; ***p-value < 0.001; NS, not significant

Mice harbouring the Eµ-*Myc* transgene display abnormal B-cell development, characterized by an initial premalignant phase in young mice (4–6 weeks) of enhanced B-cell progenitor proliferation and growth before eventually progressing to malignant lymphoma^54–56^. Both premalignant and malignant Eµ-*Myc* B-cells have elevated rRNA synthesis rates compared to wild-type B cells of the same developmental stage due to elevated expression levels of the MYC oncogene^37^. To investigate the status of rDNA chromatin with respect to rRNA transcription rates during malignant progression, we compared their dynamics in wild type, premalignant, and malignant cells isolated from Eµ-*Myc* mice. We separated these changes from variables associated with differentiation status and tumor compartment by performing analyses on IgM-negative pre-B cell subtypes (B220^high^IgM^low^IgD^low^) FACS sorted from bone marrow (with individual animals as biological replicates). MYC overexpression induced a robust increase in rRNA transcription rates in premalignant cells compared to cells from wild-type littermates, with no further significant increase observed between premalignant and malignant cells as measured using qRT-PCR (Fig. 1a) or rRNA fluorescence in situ hybridization (FISH) (Fig. 1b). We assayed rDNA chromatin structure by performing psoralen crosslinking followed by southern blot analysis. Psoralen preferentially incorporates into more accessible DNA regions and distinguishes between active (i.e., transcriptionally competent open rDNA chromatin) and inactive (i.e., silent and pseudo-silent) rRNA genes^11^. In contrast to the elevated rRNA transcription rates in both premalignant and malignant cells (Fig. 1a, b), the fraction of active rDNA repeats was significantly elevated in malignant cells only (malignant, 38.2% active vs. premalignant, 16.6% active) (Fig. 1c, d, Supplementary Fig. 1a-d). Furthermore, high ratios of active to inactive rDNA chromatin were uniformly observed in lymphoma cell lines established from independent Eµ-*Myc* tumors (lymphoma lines average, 42.8% active) as compared to wild type and premalignant cells (Fig. 1d, e). Thus, changes in the ratio of active to inactive rDNA repeats specifically occur as cells transition from premalignant to malignant and are uncoupled from MYC-driven rRNA transcription rates during malignant progression.

Loading of the Pol I architectural factor UBTF (consisting of two polypeptides, UBTF1 and 2) onto rDNA is required for the formation and maintenance of active, transcriptionally competent rDNA^12,17,57^. Therefore, we used quantitative ChIP to determine UBTF enrichment at rDNA in all stages of Eµ-*Myc* pre-B cells. Consistent with the psoralen results, UBTF enrichment was significantly increased in malignant cells compared to premalignant cells (Fig. 1f), with a modest increase in premalignant cells over wild type. These results suggest that UBTF binding at rDNA mediates the conversion of rDNA to the active chromatin state as cells transition from premalignancy to malignancy and this occurs in the absence of further increase in the rRNA transcription rate.

While CpG dinucleotide methylation of the rDNA promoter core region is implicated in silencing of murine rDNA^14,58^, we previously demonstrated that UBTF regulates the transition from pseudo-silent to open rDNA chromatin in the absence of this methylation^12,13^. To investigate the methylation status at the rDNA promoter compared to the rDNA chromatin state, we performed methylation-sensitive digestion at the −143 CCGG *Hpa*II site of genomic DNA from wild type, premalignant, and malignant cells followed by qPCR with primers spanning this site and the adjacent −133 CpG methylated residue at the rDNA promoter^12,59^. We detected no significant change in methylation at the −143 CpG (Fig. 1g, left), despite the striking shift in rDNA chromatin status during malignant progression (Fig. 1c, d). Similarly, we did not identify any significant changes in enrichment of CpG methylation at amplicons spanning the rDNA unit between cell types using quantitative methylated DNA immunoprecipitation (qMeDIP) (Fig. 1h). These findings indicate that the switch from inactive to active rDNA state during the transition to malignancy does not correlate with changes in rDNA CpG methylation. Because UBTF binding is associated with active rDNA repeats, we examined the methylation status of UBTF-bound regions by performing UBTF ChIP followed by *Hpa*II digestion and qPCR at the −143 CpG site (ChIP-CHOP), and found that the majority of UBTF-bound rDNA (~80%) was unmethylated in all cell stages (Fig. 1g, right). Collectively, these results demonstrate that the MYC-driven premalignant state, compared to wild type, is characterized by increased rRNA production without major alterations in the rDNA chromatin state. In contrast, the transition to malignancy is accompanied by the UBTF-associated activation of pseudo-silenced rDNA (termed herein rDNA class switching), which is uncoupled from changes in rRNA transcription rates.

### Eµ-*Myc* lymphoma survival requires UBTF-mediated active rDNA

**Fig. 2:**
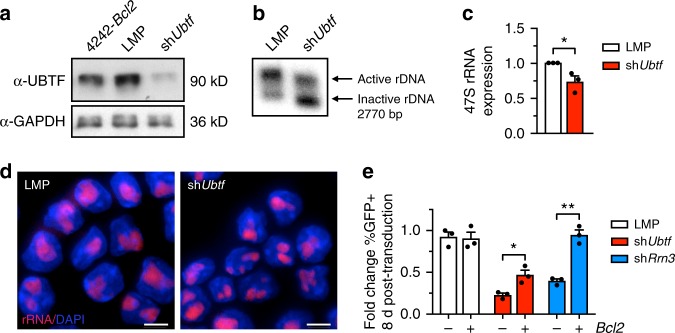
UBTF loss in lymphoma cells reduces active rDNA and cell survival independent of rDNA transcription. **a** Eμ-*Myc*-*Bcl2* cells (4242-*Bcl2)* were stably transfected with empty vector LMP (Eμ-*Myc*-LMP cells) or sh*Ubtf* (Eμ-*Myc*-sh*Ubtf* cells) and *Ubtf* knockdown verified by Western blot of UBTF protein compared to GAPDH loading control. **b** Representative Southern blot of psoralen cross-linked genomic rDNA from Eμ-*Myc*-LMP and Eμ-*Myc*-sh*Ubtf* cells as in Fig. 1c. **c** Relative pre-rRNA (47SrRNA) expression in Eμ-*Myc*-LMP or Eμ-*Myc*-sh*Ubtf* cells determined by qRT-PCR. Pre-rRNA levels were determined as in Fig. 1a, at 72 h post transduction. RNA levels were normalized to *B2M* mRNA and are represented relative to Eμ-*Myc*-LMP ($\bar x$ ± s.e.m., **p-value < 0.01; *n* = 3). **d** rRNA levels in Eμ-*Myc*-LMP or Eμ-*Myc*-sh*Ubtf* cells determined by rRNA FISH as in Fig. 1b. Scale bars, 10 μm. **e** GFP competition assay using Eμ-*Myc*-LMP, Eμ-*Myc*-sh*Ubtf*, and Eμ-*Myc*-sh*Rrn3* (sh*Rrn3*) cells co-cultured with mock-transduced Eμ-*Myc* cells either in the absence (−) or presence (+) of *Bcl2* overexpression. Data are expressed as percent fold change in GFP+ cells 8 days post transduction ($\bar x$ ± s.e.m., *p-value < 0.05; **p-value < 0.01; *n* = 3)

Given the observation that clonal Eμ-*Myc* lymphomas show significantly elevated ratios of active rDNA repeats, we hypothesised that rDNA class switching is favorable to the malignant state. To test this, we investigated whether tumor cell survival is dependent on the elevated active:inactive rDNA ratio independent of rRNA transcription rate. We previously established that *Ubtf* knockdown leads to a reduction in the number of active rDNA repeats without affecting overall rRNA transcription rate due to compensatory mechanisms that maintain rRNA synthesis at a constant level^12^. Here, we confirmed this observation using a shRNAmiR targeting *Ubtf* (*Ubtf1/2*)^37^ in an Eμ-*Myc* lymphoma cell line (Eμ-*Myc*-sh*Ubtf*). Knockdown of *Ubtf* led to a robust reduction of UBTF compared to control cell line (Eμ-*Myc*-LMP) (Fig. 2a) that was accompanied by a pronounced reduction in active rDNA repeats (Fig. 2b) but only a modest reduction in relative rRNA transcription rates (Fig. 2c, d).

To investigate whether the reversal of rDNA class switching by *Ubtf* knockdown impacts cell proliferation and survival, we used an in vitro GFP-based cell competition assay^37^. Mock-transduced Eμ-*Myc* lymphoma cells were seeded in culture with equal numbers of cells retrovirally transduced with either empty vector or sh*Ubtf* expressing vector, and survival of the vector-driven GFP expressing cells was monitored by FACS. Long-term (8 days) competition revealed that sh*Ubtf-*expressing cells have an acute survival disadvantage compared to control cells (Fig. 2e). Moreover, this survival handicap was only partially rescued by overexpression of the anti-apoptotic factor BCL2. In comparison, we performed knockdown of the Pol I transcription initiation factor RRN3 (sh*Rrn3*)^37^, which leads to reduced rRNA transcription but does not regulate rDNA chromatin remodelling^57^. *Rrn3* knockdown also resulted in a proliferation disadvantage compared to control cells, but unlike *Ubtf* knockdown, the cell death in response to RRN3 loss was completely rescued by BCL2 overexpression (Fig. 2e). This is consistent with our previous data showing that Eμ-*Myc* lymphoma cells treated with CX-5461, a highly selective inhibitor of Pol I-mediated transcription^36^, are over 100-fold less sensitive to apoptotic cell death following BCL2 overexpression^37^. CX-5461 inhibits rDNA transcription by preventing recruitment of the transcription competent Pol I complex (including RRN3) while minimally perturbing UBTF association with the rDNA^51^. Following treatment of lymphoma cells with CX-5461, we observed no significant change to the ratio of active to inactive rDNA despite repression of rRNA transcription (Supplementary Fig. 2). Taken together, these data indicate that survival of malignant Eμ-*Myc* cells is dependent on the ability of UBTF to maintain an active rDNA chromatin state independent of the rRNA transcription rate.

### rDNA repeats engage in long-range chromatin interactions

**Fig. 3:**
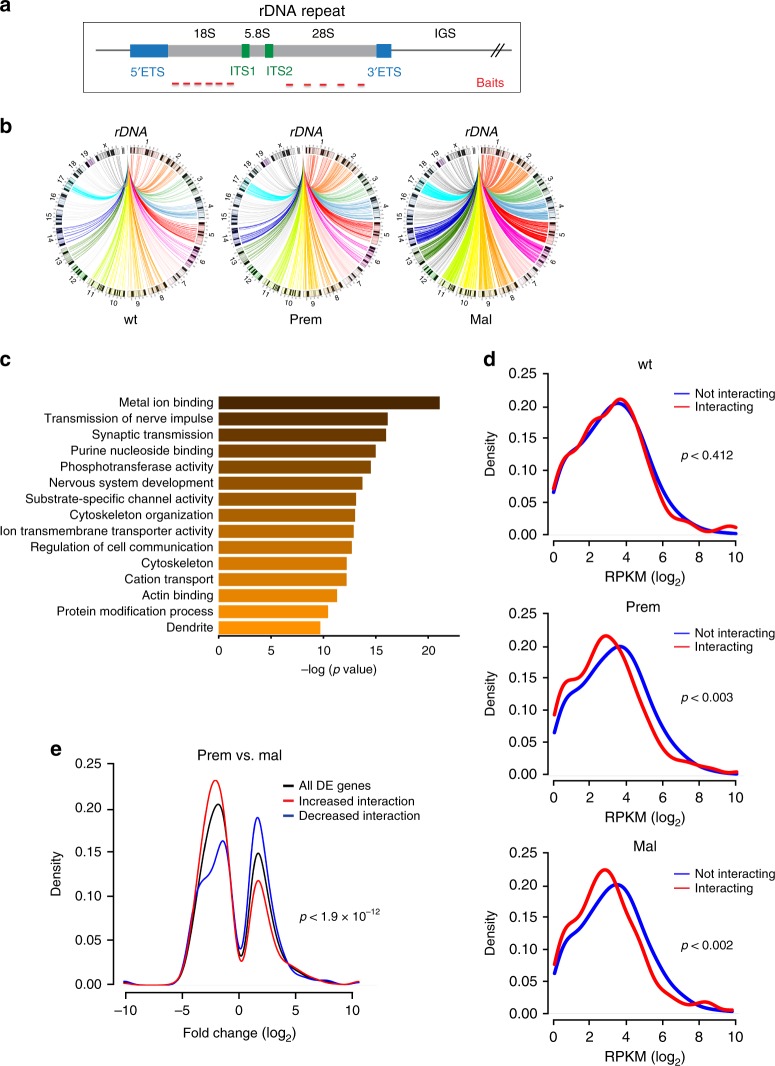
rDNA repeats engage in long-range chromatin interactions during lymphomagenesis. **a** Schematic representation of a representative rDNA repeat indicating the 4C-seq bait positions sequences listed in Supplementary Table 1). **b** Circos plots illustrating the top 10% significantly differential interactions (between a representative 28S rDNA bait and the rest of the genome) in wild type (wt), premalignant (prem) and malignant (mal) Eμ-*Myc* cells. The depicted differences in rDNA interactions between cell stages is qualitative only. Interactions with each chromosome are highlighted in a different color. **c** GO analysis of protein coding genes in regions constitutively interacting with rDNA between wild type, premalignant, and malignant cells. Constitutively interacting regions associated with protein coding genes (9382 unique genes, from Supplementary Data 2) were subjected to gene set over-representation analysis using ConsensusPathDB (mouse) to identify enriched GO terms^90–92^, with *p* values referring to enrichment of the terms in the described dataset. The genes contributing to these GOs include those encoding zinc finger domain, transmembrane channel and receptor proteins. *p*-value (x-axis) denotes the significance of the number of interacting genes compared to the total number of genes in the GO classification. **d** Density histograms of gene expression (RPKM) distributions for genes associated with regions that do interact (red line) and do not interact (blue line) with the rDNA in wild type, premalignant and, malignant cells. Significance between groups was assessed using the nonparametric Kolmogorov-Smirnov (K-S) test. **e** Density histogram of fold change (log2) in differentially expressed (DE) genes (premalignant vs. malignant) for all genes (including not interacting, black line) and genes associated with significantly increased interactions (red line) and significantly decreased interactions (blue line) with the rDNA in malignant vs. premalignant cells. Significance between increased and decreased interactions was assessed using the K-S test

The observation that the rDNA chromatin status affects tumor cell survival independent of rRNA transcription rate per se suggests that changes in rDNA chromatin might impart functional effects. Non-rDNA-genomic regions, called NADs, localize to the nucleolus, which forms around actively transcribed rDNA repeats^24–28^. Changes in rDNA structure/activity can influence genome-wide chromatin structure and gene expression^19,20^, therefore we examined whether rDNA class switching promotes reorganization of NADs. We used 4C-seq to identify long-range chromatin interactions that occur with rDNA sequences during lymphomagenesis. Multiple bait sequences located in the transcribed 18S and 28S rDNA regions (Fig. 3a) were used to capture interactions between the rDNA and rest of the genome in isolated wild type, premalignant, and malignant pre-B cells. Cell-type specific rDNA interactions were included in subsequent analyses if they were observed in two biological replicates (i.e., animals). We combined all replicated rDNA interactions for each cell type and calculated the average read count per interaction in 5-kb non-overlapping windows as a semi-quantitative measure of interaction frequency (read counts listed in Supplementary Data 1). The interactions were not restricted to a few specific loci and did not show a bias towards the rDNA-containing chromosomes (12, 15, 18, and 19 in the Eμ-*Myc* C57BL/6 genetic background^60^), but were uniformly distributed across all chromosomes (Supplementary Fig. 3a). We categorized the rDNA-genome interactions into two categories: those that show no significant change (FDR > 0.1) between wild type, premalignant, and malignant cells (constitutive rDNA interactions, Supplementary Data 2); and those that are significantly changed (FDR < 0.1) between any pair of cell stages (differential rDNA interactions). We plotted the top 10% of differential rDNA interactions (regardless of fold-change direction) and while qualitative only, circos plots revealed that the major overall interaction re-configuration occurs between premalignant and malignant cells (Fig. 3b). These results suggest that B-cell lymphomagenesis is associated with changes in the pattern of interactions that rDNA forms with the rest of genome.

### rDNA-genome interactions correlate with transcriptome changes

Specific gene families are reported to be enriched within NAD sequences^24,25,27^, therefore we sought to characterize whether this occurred during malignant progression. We determined the closest gene for each rDNA-interacting fragment based on the nearest transcription start site (TSS). Analysis of gene ontologies (GOs) of protein coding genes associated with constitutive rDNA-interacting regions showed an over-representation of GO terms that comprise zinc finger domain proteins, transmembrane channel proteins and G-protein-coupled receptors (Fig. 3c, Table 1 and Supplementary Data 2). Inspection of all genes mapping to constitutively interacting regions revealed an enrichment of non-coding RNA (ncRNA) genes (e.g., small nucleolar RNAs (snoRNAs), spliceosomal small nuclear RNAs (snRNAs)) and genes encoding olfactory and vomeronasal receptors (Table 1, Supplementary Data 2). These results are in keeping with previous studies demonstrating nucleolar enrichment of specific gene classes including certain ncRNAs and zinc finger and olfactory receptor encoding genes^24,25,27^. Interestingly, while we detected 5S rRNA pseudogenes within the constitutive interaction set, we did not detect any rDNA interactions with the canonical 5S rRNA array on chromosome 8, consistent with reported Hi-C data^28,61^.

**Table 1:** Constitutive rDNA-genome interactions between all cell states and associated genes (GO term enrichment, Fig. 3c)

Window	Mean norm counts: wild type	Mean norm counts: premalignant	Mean norm counts: malignant	Gene name	Gene description (GO term)
chr8:111250001–111255000	76.8	66.5	80.2	*Zfhx3*	zinc finger homeobox (metal ion binding)
chr7:87180001–87185000	73.7	70.5	55.5	*Zfp710*	zinc finger protein (metal ion binding)
chr2:62840001–62845000	99.7	61.5	72.0	*Kcnh7*	potassium voltage-gated channel (substrate-specific channel activity)
chr11:48850001–48855000	62.3	70.5	77.1	*Olfr56*	olfactory receptor
chr15:95775001–95780000	64.4	95.3	86.4	*Ano6*	Ca2+ activated chloride channel (ion transmembrane transporter activity)
chr8:41560001–41565000	72.7	52.6	80.2	*Zdhhc2*	zinc finger, DHHC-type containing (protein modification process)
chr7:49120001–49125000	159.9	170.7	186.1	*Vmn2r58*	vomeronasal receptor
chr6:136000001–136005000	83.0	92.3	72.0	*Grin2b*	NMDA ionotropic glutamate receptor (transmission of nerve impulse)
chr1:87570001–87575000	76.8	85.3	97.7	*n-R5s215*	nuclear encoded rRNA 5S 215
chr12:92015001–92020000	76.8	63.5	126.5	*n-R5s65*	nuclear encoded rRNA 5S 65
chr13:20960001–20965000	145.3	127.0	170.7	*Gm25605 (U1)*	U1 spliceosomal snRNA
chr15:94530001–94535000	174.4	229.2	232.3	*SNORA17*	Small nucleolar RNA SNORA17
chr17:88855001–88860000	152.6	158.8	164.5	*snoU13*	Small nucleolar RNA U13

The nucleolar periphery is characterized by a facultative heterochromatic shell and transcriptionally inactive regions^30^, thus we determined if there is an association between physical interactions with the rDNA and gene expression. We performed RNA-seq on wild type, premalignant, and malignant cells and determined the average gene expression (reads per kilobase of transcript per million reads mapped [RPKM]) for all genes (Supplementary Data 3). We compared the density of RPKM distributions for genes associated with rDNA-interacting (constitutive and differential) and non-interacting regions for all stages of malignant progression (Fig. 3d). Genes associated with rDNA-interacting regions (Fig. 3d, red lines) in the premalignant and malignant cells, but not wild-type cells, had significantly lower expression levels than non-interacting genes (Fig. 3d, blue lines). This observation is consistent with previous findings^24–27^ that association with the rDNA correlates with a repressive effect on gene expression.

Global gene expression analysis identified 4908 differentially expressed genes during malignant progression (wild type vs. malignant cells, FDR ≤ 0.1; −0.5 ≥ logFC ≥ 0.5) (Supplementary Data 3). We intersected these with the 23,476 rDNA interactions that changed during progression from premalignant to malignant, coincident with rDNA class switching (Supplementary Data 4). The majority of differentially expressed genes (3961 out of 4908 genes) were proximal to differentially interacting regions. Notably, we observed a robust inverse relationship between rDNA interaction and gene expression for rDNA-interacting genes whose expression changes significantly from premalignant to malignant (746 genes) (Fig. 3e). These results showed a correlation between interaction with the rDNA and transcriptional repression of associated genes as cells transition from premalignancy to malignancy, suggesting that rDNA-NAD interactions contribute to Pol II gene regulation during the development of malignancy.

### UBTF-dependent interactions involve specific gene pathways

**Fig. 4:**
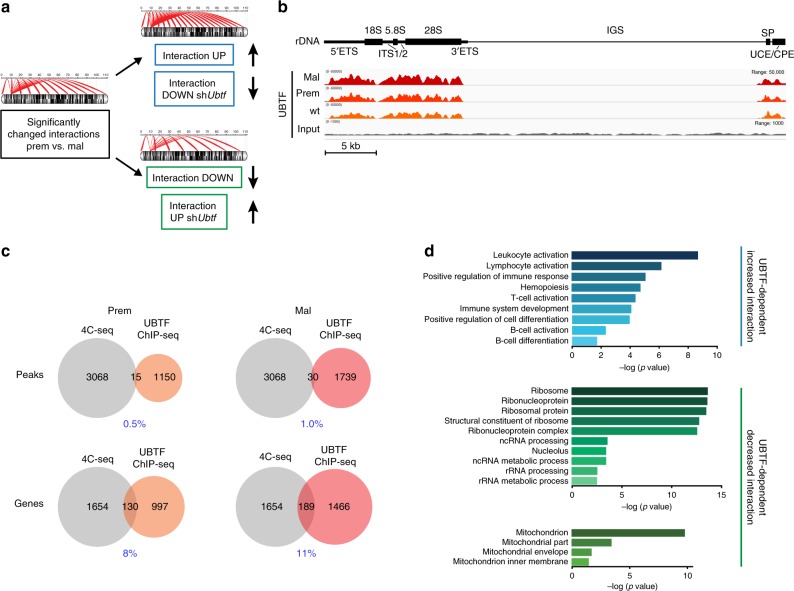
UBTF-dependent rDNA class switch interactions involve specific gene pathways. **a** Schematic illustrating the filtering workflow for identification of UBTF-dependent rDNA-genome interactions that change from premalignant (prem) to malignant (mal) cells. Interactions that were not UBTF-dependent or not identified in Eμ-*Myc*-LMP or Eμ-*Myc*-sh*Ubtf* cells were removed. **b** Integrated Genome Viewer screenshots of UBTF ChIP-seq enrichment at the mouse rDNA repeat (one replicate shown), indicated by the schematic (top); ETS and ITS, external and internal transcribed spacers, respectively; IGS, intergenic spacer; SP, spacer promoter; UCE, upstream control element; and CPE, core promoter element, along with the 18S, 5.8S, and 28S sequences depicted. Below the schematic are ChIP-seq tracks from wild type (wt), premalignant, and malignant cells (set to display the same data range) with the input control track shown at the bottom (data range adjusted for visualisation) and scale depicted underneath. **c** Intersection of UBTF-dependent rDNA-NAD interactions with UBTF ChIP-seq. Venn diagrams indicating the overlap of UBTF-dependent rDNA-interacting regions (selected as shown in (**a**)) with UBTF binding sites (top) and nearest associated unique genes for both sets of regions (bottom) in premalignant and malignant cells. **d** The enriched GO terms were identified using standard DAVID analysis^93,94^, with *p* values referring to enrichment of the terms in the described datasets as in Fig. 3c. These enriched ontologies include B-cell differentiation in UBTF-dependent increased interactions or ribosome metabolism and energy metabolism ontologies in UBTF-dependent decreased interactions

To identify interactions that are reliant upon UBTF-dependent rDNA class switching as opposed to those that are intrinsically a consequence of malignant transformation, we performed 4C-seq in the stable *Ubtf* knockdown lymphoma cells (Eμ-*Myc*-sh*Ubtf*) compared to control cells (Eμ-*Myc*-LMP). Again, the distribution of the *Ubtf-*dependent rDNA interactions (Supplementary Data 5) was spread across all chromosomes (Supplementary Fig. 3b). rDNA interactions with genomic DNA in mouse embryonic stem cells (mESCs) have been reported using the recently developed split-pool recognition of interactions by tag extension (SPRITE) method^62^. We interrogated the overlap of mESCs SPRITE rDNA contacts with our 4C-seq rDNA interactions identified in the Eμ-*Myc* lymphoma cell lines. This analysis indicated a strong concurrence between the SPRITE-identified rDNA hub genomic connections and our 4C rDNA-NAD connections for both the LMP and sh*Ubtf* conditions, with bootstrap samples confirming the non-random nature of the intersection (Supplementary Fig. 3c). We intersected the rDNA interactions detected in the *Ubtf* knockdown cells with those from the Eμ-*Myc* pre-B cells and identified reciprocal interactions that either increase upon premalignant to malignant transition and decrease with UBTF loss (1822 interactions) or decrease upon premalignant to malignant transition and increase with UBTF loss (1246 interactions) (Fig. 4a, Supplementary Data 6). These UBTF-dependent rDNA class switch interactions correlated with the changes in rDNA chromatin state that occur during transition to malignancy and are reversed by UBTF knockdown (Fig. 4a).

A substantial fraction (28%: 864 of 3068 total interactions) of the rDNA class switch interacting regions were located within 5–20 kb of the associated gene’s TSS (Supplementary Fig. 4a). Considering that changes in rDNA interaction correlated with altered expression levels of the nearest gene (Fig. 3e), we examined whether these rDNA-interacting fragments are enriched for putative regulatory elements by intersecting them with regions marked by the enhancer-associated H3K4me1 histone modification in Eμ-*Myc* tumor cells^63^. Thirteen percent of loci that differentially interacted with the rDNA during the premalignant to malignant transition were located in H3K4me1-enriched peaks, and this increased two-fold to 27% (835 of 3,068 interactions) when we intersected UBTF-dependent rDNA class switch interactions only (Supplementary Fig. 4b). H3K4me1 and H3K4me3 are co-located at transcriptionally poised and/or active genes^64^. Therefore, we intersected the H3K4me1 overlapping rDNA class switch regions with identified H3K4me3 enriched regions^63^. The majority (66% of 835) of the UBTF-dependent rDNA class switch regions were enriched in H3K4me1 only, while the remaining 34% were enriched for both H3K4me1 and H3K4me3 (Supplementary Fig. 4b). In addition to its roles in regulating Pol I transcription and rDNA chromatin status, UBTF has also been shown to directly regulate a sub-fraction of Pol II gene transcription^65^. Thus, we performed UBTF ChIP-sequencing in the malignant progression setting to determine whether UBTF is enriched at UBTF-dependent rDNA class switch interaction regions. As previously reported^65,66^, the majority of UBTF enrichment occurred at the promoter and transcribed sequences of the rDNA repeat (Fig. 4b). We intersected non-rDNA repeat UBTF-enriched peaks from premalignant and malignant cells (Supplementary Data 7) with the corresponding UBTF-dependent rDNA interaction regions and found only ≤1% overlap (Fig. 4c). However, when we intersected the nearest-located genes to UBTF peaks with those associated with UBTF-dependent rDNA-interacting regions, we observed an ~10% overlap of genes in both premalignant and malignant cells (Fig. 4c, Supplementary Data 7). While this degree of overlap might, at least in part, reflect chance occurrence, it nonetheless suggests the potential for UBTF loss to directly impact expression of a small subset of rDNA class switch associated genes.

We performed GO analysis on the genes associated with rDNA class switch interactions that exhibited either increased rDNA interaction/decreased gene expression or conversely, decreased rDNA interaction/increased gene expression (wild type to malignant) during malignant transformation. B-cell differentiation and lineage specification ontologies—including genes such as *Ebf1*, *Pbx1*, and *Runx1*—were significantly enriched in the regions that exhibited UBTF-dependent increased interaction and decreased expression (Fig. 4d, Table 2 and Supplementary Data 8). This observation is consistent with the established role of compromised B-lineage-specific gene function in hematologic malignancies via impaired differentiation^67,68^. Furthermore, the regions that displayed UBTF-dependent decreased interaction and increased expression were enriched in genes associated with ribosome function, RNA processing/metabolism and mitochondrial/energy metabolism processes, e.g., *Parn*, *Eif3d, Dhodh*, and *Pdp2* (Fig. 4d, Table 3 and Supplementary Data 8). Both RNA processing and mitochondrial function pathways are transcriptionally regulated by oncogenic MYC and dysregulated during malignancy^41,42^, and are consistent with GOs that were recently identified as being enriched in rDNA Hi-C contacts detected in human lymphoblastoid and leukemic cell lines^69^. These findings reinforced the concept that UBTF-dependent changes in rDNA interaction are associated with altered gene expression, potentially through enhancer sequences located within the rDNA-interacting fragments and, in a minor subset, through direct UBTF regulation. Collectively, these results are consistent with a model in which reformed rDNA-NAD interactions that occur with UBTF-driven changes in rDNA chromatin during the transition to B-cell malignancy coincide with gene expression changes that impact specific functional pathways that promote the malignant phenotype.

**Table 2:** Increased rDNA class switch interacting regions (window) in malignant compared to premalignant cells corresponding to B-cell differentiation genes (decreased expression from wild type to malignant cells)

Window	LogFC interaction	LogFC expression	Gene name
chr16:35870001–35875000	2.829	−1.198	*Parp14*
chr11:33930001–33935000	1.892	−4.911	*Lcp2*
chr16:19925001–19930000	1.250	−1.850	*Klhl6*
chr11:44660001–44665000	0.909	−1.925	*Ebf1*
chr16: 92765001–92770000	1.514	−1.504	*Runx1*
chr11:44790001–44795000	0.443	−3.657	*Il7r*
chr19:34395001–34400000	0.751	−2.090	*Fas*

**Table 3:** Decreased rDNA class switch interacting regions (window) in malignant compared to premalignant cells corresponding to RNA metabolism or energy metabolism genes (increased expression from wild type to malignant cells)

Window	LogFC interaction	LogFC expression	Gene name
chr14:55520001–55525000	−1.734	0.851	*Pabpn1*
chr17:56755001–56760000	−0.758	1.771	*Rpl36*
chr6:71885001–71890000	−0.719	2.377	*Polr1a*
chr15:77855001–77860000	−0.662	1.415	*Eif3d*
chr8:107125001–107130000	−2.623	0.980	*Pdp2*
chr8:112115001–112120000	−0.657	1.402	*Dhodh*
chr1:60380001–60385000	−0.118	1.621	*Cyp20a1*
chr3:97415001–97420000	−3.280	1.570	*Chd1l*
chr3:132755001–132760000	−1.026	1.770	*Gstcd*

### rDNA-interacting loci undergo nuclear re-localization

**Fig. 5:**
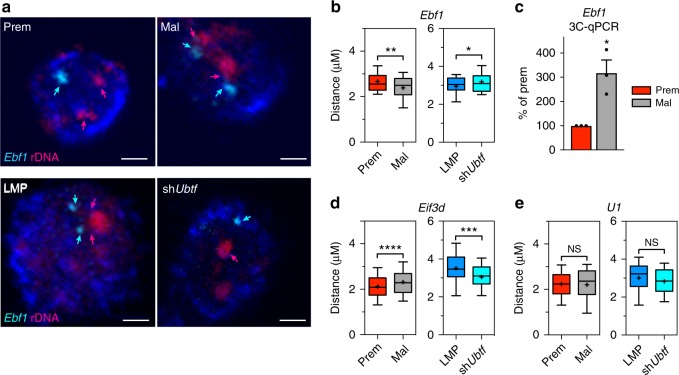
rDNA class switch-dependent interacting loci undergo nuclear re-localization. **a** Representative 2D-FISH images taken from the 3D-FISH Z stack images of nuclei depicting a decrease in distance between the early B cell factor 1 (*Ebf1*) gene (cyan, Alexa 488 signal; cyan arrows) and the rDNA (magenta, Alexa 555 signal; magenta arrows) between premalignant (prem) and malignant (mal) cells. The distance decrease is reversed from LMP (Eμ-*Myc*-LMP) to sh*Ubtf* (Eμ-*Myc*-sh*Ubtf*) cells. Nuclei were counterstained with DAPI (blue). Scale bars, 2 μm. **b** Boxplots of distances measured from 3D-FISH analysis between the rDNA and the *Ebf1* locus in premalignant (red) compared to malignant (gray) cells and LMP (blue) compared to sh*Ubtf* cells (cyan). Distances between the closest two foci in each nucleus were measured using the Volocity software. Whiskers correspond to the 10th and 90th percentiles of the data range, with data points outside this range not depicted. Significance was assessed using the Wilcoxon–Mann–Whitney test (*p-value < 0.05, **p-value < 0.01, NS = not significant, *n* > average 170 distances). **c** 3C-qPCR validation of the increased interaction frequency between the *Ebf1* locus and the rDNA in malignant compared to premalignant cells. The interaction frequency was normalised to a single copy locus (*Ercc3* gene) and a multi copy locus (28S rDNA) and is represented as interaction frequency relative to premalignant cells (% of premalignant) ($\bar x$ ± s.e.m., *p-value < 0.05; *n* = 3). Boxplots of distances measured from 3D-FISH analysis between the rDNA and the **d** eukaryotic translation initiation factor 3, subunit D (*Eif3d*) locus between premalignant and malignant cells (increased distance) and reversed with *Ubtf* knockdown; and the **e** constitutively interacting *U1* spliceosomal snRNA (official MGI gene symbol, Gm25605) locus as described for **b** (***p-value < 0.001, ****p-value < 0.0001, *n* > average 170 distances)

Our results demonstrated that specific loci undergo altered interactions with rDNA during malignant progression, which is associated with differential gene expression (Fig. 3e). Such changes in interaction frequency are likely the result of physical changes in the relative positions of rDNA and the locus in question. To test this, we measured the center-to-center distances between representative interacting loci and the rDNA using dual-labeled 3D DNA-FISH (3D-FISH)^70^ (Fig. 5, Supplementary Fig. 5). The rDNA FISH specificity was validated by comparing its localization with that of the rDNA-associated pre-rRNA processing protein Fibrillarin (FBL) (Supplementary Fig. 5a). We measured the distance between rDNA and *Ebf1*, a gene identified by 4C-seq to have increased rDNA interaction in malignant vs. premalignant cells, which is decreased upon *Ubtf* knockdown in malignant cells. As predicted, the distance between *Ebf1* and rDNA decreased in malignant compared to premalignant cells (representative images Fig. 5a, top; quantitation Fig. 5b, left) while conversely, the distance increased in Eμ-*Myc*-sh*Ubtf* compared to control cells (Eμ-*Myc*-LMP) (Fig. 5a, bottom; Fig. 5b, right). The significant increase in interaction frequency (decreased distance) between the *Ebf1* locus and rDNA during malignant progression was further validated by 3C-qPCR (Fig. 5c). We also confirmed the opposite 4C-seq interaction pattern, with 3D-FISH showing that the distance between the *Eif3d* locus and rDNA increased during the premalignant to malignant transition and decreased with UBTF knockdown (Fig. 5d; Supplementary Fig. 5b shows additional 3D-FISH assayed loci: *Cyp51*, *Parn*, *Chd1l*; Supplementary Fig. 5c shows additional 3C-qPCR assayed loci: *Pdp2* and *Pbx1*). 3D-FISH also confirmed that no significant change in distance was measured between the *U1* small spliceosomal RNA gene and the rDNA in either cell condition (Fig. 5e), consistent with its constitutive association with the rDNA. Notably, some of the validated relocalized genes (e.g. *Ebf*, *Pbx1*) were also associated with UBTF enrichment (Supplementary Data 7). Taken together, these results demonstrate that malignant progression coincides with a subset of specific loci that are ontologically associated with the malignant phenotype undergoing a concurrent program of changes in physical localization, rDNA interaction and gene expression, and that these changes directly correlate with UBTF-mediated rDNA class switching.

## Discussion

We demonstrate that the rDNA chromatin state is decoupled from rRNA transcription during the progression from premalignancy to malignancy through a process of pseudo-silenced rDNA activation that we term rDNA class switching. This observation is consistent with the demonstration that rDNA chromatin status does not strictly correlate with rRNA transcription rates^12,13^. Indeed, complete loss of UBTF via conditional deletion and subsequent eradication of the active rDNA fraction is required before a significant impact on rRNA transcription is observed^17^. Interestingly, the occurrence of rDNA class switching during MYC-driven lymphomagenesis mirrors the reverse observation as granulocytic cells undergo differentiation accompanied by a reduction in MYC, which regulates *UBTF* expression^12,13,44^. We speculate, given that MYC is dysregulated in upwards of 70% of human cancers^41,71^, that rDNA class switching is a more general mechanism underlying malignant transformation.

Concomitant with rDNA class switching we observed a marked repatterning of rDNA-NAD interactions. While chromosome conformation capture techniques have previously identified genomic regions associated with the rDNA^28,62,72^, the potential dynamic regulation of rDNA-genome contacts and their functional significance for disease are not well understood. Here we demonstrate that as preneoplastic cells become malignant, changes in the rDNA-interactome correlate with changes in proximal gene expression, an observation supported by previous reports that established a link between NAD-associated genes and transcriptional repression^24–27^. Importantly, our findings identify a subset of altered rDNA-interacting loci and their associated gene expression changes that are dependent on UBTF-mediated rDNA class switching. Elucidation of this dynamic rDNA interaction network in the Eµ-*Myc* cancer model revealed that rDNA class switching is associated with functional consequences for programs of Pol II transcriptional activity that are relevant to malignant cell fitness.

rDNA class switching during the malignant transition is accompanied by a significant increase in UBTF loading onto the rDNA. We and others have shown that UBTF binding correlates with psoralen accessibility^12,17,57,73^; however, UBTF alone is unlikely sufficient to drive rDNA class switching. Specifically, *Ubtf* knockdown does not significantly alter repressive chromatin modifications and enforced *Ubtf* expression induces only a modest increase in the proportion of active rDNA^12^. These observations suggest that prior to association of UBTF, additional epigenetic modifications occur at the rDNA that are required to facilitate UBTF enrichment and rDNA class switching in malignant cells.

We recently reported a link between UBTF and Pol II gene transcription by identifying a role for UBTF at the promoters of highly Pol II-transcribed genes^65^. Our genome-wide analysis of UBTF enrichment in Eµ-*Myc* cells showed minimal overlap (≤1%) between UBTF-bound (non-rDNA) sequences and the rDNA-interacting regions that require UBTF (Fig. 4c). Moreover, the rDNA class switch regions do not intersect with previously identified UBTF-bound regions such as the histone gene clusters^65^. However, when we extend the analysis to the nearest associated genes, we observe that ~10% of these rDNA-interacting loci are potentially regulated by UBTF. While further investigation is required, these data suggest that UBTF does not regulate Pol II genes directly through rDNA-interacting regions, but may influence the expression of a small subset of the UBTF-dependent rDNA-interacting genes either via proximity while bound to rDNA or enrichment at these genes exclusive of rDNA. Notably, several of the UBTF-enriched Pol II genes (Supplementary Data 7) were validated by 3D-FISH and/or 3C-qPCR to relocalize with respect to the rDNA during malignant progression (e.g., *Ebf1* and *Pbx1*). Thus, we propose that a complex regulatory network including rDNA interactions and, in some instances, direct UBTF regulation of Pol II gene transcription, contributes to gene expression reprogramming during malignant progression. Nonetheless, silencing *Ubtf* as a means to reverse rDNA class switching revealed that the majority of interactions rely on the altered rDNA state independent of UBTF enrichment. Ongoing studies are focused on identifying factors that directly mediate these dynamic rDNA interactions during malignant transformation. The NoRC subunit TIP5/BAZ2A and MYC mediate interactions between the rDNA and the nuclear matrix^74,75^ while the chromatin regulatory factor CTCF interacts with rDNA and is proposed to facilitate UBTF loading onto rDNA, thereby contributing to the activation of silent rDNA repeats^57,66,72,76,77^. The observed enrichment of the enhancer-associated histone H3K4me1 modification at rDNA class switch-dependent regions associated with altered gene expression suggests a gene regulatory function for these interactions. Ultimately, a comprehensive interrogation of these genomic loci via their genetic disruption and the identification of their interacting protein components will be required to elucidate their functional significance.

MYC overexpression drives the Eμ-*Myc* premalignant phenotype, which we have characterized by increased rRNA transcription of existing active rDNA repeats. We propose that subsequent epigenetic events during the transition to malignancy result in increased UBTF association with rDNA and rDNA class switching. The question of whether the captured genomic interactions occur with the active rDNA repeats only or also the silent rDNA remains. Technical challenges preclude our ability to directly address this question. However, it is predicted that the malignancy-associated pronounced increase in active rDNA coincides with altered physical properties of the rDNA chromatin. Recent studies implicate a role for the biophysical properties of phase separation between sub-nuclear compartments leading to the nucleolar sequestration of certain proteins and driving functional heterochromatin formation^78–80^. Another intriguing consideration is that the epigenetic and/or transcriptional activity of the rDNA might impact the transcriptional state of the interacting genomic regions through titration of epigenetic modifying or Pol II transcription factors.

Our results provide compelling evidence that the formation of specific rDNA interactions contributes to transcriptional reprogramming of functionally relevant gene pathways. For example, genes associated with increased rDNA interaction and decreased expression during malignancy are enriched for B-cell lineage and differentiation genes, i.e., pre-B cell leukemia homeobox 1 (*Pbx1*), Early B-cell Factor 1 (*Ebf1*), and *Runx1*. The dysregulation of lineage-associated genes leads to impaired differentiation in hematologic malignancies, such as acute leukemias and some lymphomas^67,68,81^. While perturbation of differentiation or lineage specification is often due to genetic alterations including sequence mutation or chromosomal translocation, our findings indicate that dysregulation of B-cell lineage genes can be influenced by relocalization of genomic regions such as NADs. The demonstration that a developmentally orchestrated nuclear re-localization of the *Ebf1* locus contributes to *Ebf1* transcriptional activation during B-cell differentiation^82^ corroborates such a mechanism. Our data support a model where structural changes in rDNA chromatin promote gene expression changes that influence clonal selection of a malignant cell population.

Together, our data are consistent with the notion that the rDNA repeats play a key regulatory role in cellular function during disease development, independent of their role in determining ribosome content and growth rates. We propose that this novel role is mediated through the formation and maintenance of dynamic programs of spatial genome organization that regulate distinct patterns of gene expression. Thus, developing tools to target rDNA chromatin regulatory factors (such as UBTF) might provide a therapeutic window in certain cancers. Previous studies on Pol I transcription inhibitors as cancer therapies^37,39,50,52^ suggest that there are multiple mechanisms via which rDNA transcription and chromatin may be targeted for therapeutic benefit. If so, an increased fraction of active rDNA repeats may function as a biomarker for the sensitivity of cancer cells to inhibition of rRNA transcription.

## Methods

### Animal experiments and B-cell purification strategy

All animal experiments were performed with approval from the Peter MacCallum Cancer Centre Animal Experimentation Ethics Committee. Eμ-*Myc* mice were maintained as heterozygote on a C57BL/6 background and monitored twice a week for lymphoma development. Lymphomas can arise from expansion of either B220^+^/IgM^−^ or B220^+^/IgM^+^ clones. Thus, to reduce the potential for variability in our study, we focused our analysis on the IgM-negative B-cell subtypes sorted via flow cytometry from the bone marrow niche from mice with normal B-cells, and from both premalignant and fully malignant B cells obtained from Eµ-*Myc* mice, i.e., the B220^high^IgM^low^IgD^low^ progenitor B (pro-B)/precursor B (pre-B) cells from premalignant Eµ-*Myc* mice and their wild-type littermate controls, and the B220^+^IgM^−^IgD^−^ transformed pre-B cells from mice that have developed spontaneous disease (malignant). Female mice were used for all wild type and premalignant cell collections while both female and male mice were harvested for malignant cells due to the spontaneous development of the lymphomas. Therefore, this strategy allowed us to perform our experiments largely outside of variables due to differentiation and tumor niche. Wild type and premalignant mice were sacrificed at age 4–6 weeks and malignant mice as soon as they established spontaneous lymphoma (age 10–20 weeks).

### Lymphoma cell line studies

For all in vitro experiments, the tissue cultured adapted Eμ-*Myc* clone #4242 was used and maintained in Anne Kelso DMEM supplemented with 10% fetal bovine serum (FBS), 100 mM L-asparagine (Sigma), 0.5% ß-mercaptoethanol, and penicillin/streptomycin/glutamine (GIBCO). A stable *Ubtf* knockdown (the validated sh*Ubtf* hairpin^37^ targets both UBTF1 and UBTF2 protein isoforms) cell line was generated in a 4242 Eμ-*Myc* line^37^ overexpressing BCL2 to maintain sufficient cell survival with reduced UBTF (also generated without BCL2 for GFP competition assay). Cells were retrovirally transduced with sh*Ubtf*-MSCV-LTRmiR30-PIG or empty LMP retroviral vectors (also sh*Rrn3*-MSCV-LTRmiR30-PIG for GFP competition assay; the sh*Rrn3* hairpin was also previously validated^37^) (Open Biosystems) and transduced populations selected by sorting for high GFP expression using flow cytometry. For the GFP-based cell competition assays^37^, the indicated Eμ-*Myc*-LMP, Eμ-*Myc*-sh*Ubtf,* or Eμ-*Myc*-sh*Rrn3* cells were mixed with equal numbers of mock-transduced (no vector) cells, cultured over 8 days and the percentage of GFP+ cells determined by FACS. Eμ-*Myc* lymphoma cells were treated with 50 nm CX-5461 and NaH2PO4 vehicle for 1 h prior to harvesting for psoralen cross-linking and qRT-PCR analysis.

### 3D DNA-FISH

Cells were fixed with 4% paraformaldehyde and cytospun on poly-l-lysine-coated slides. Prior to hybridization, cells were permeabilized using 0.5% Triton X-100 (20 min at room temperature), incubated in 20% glycerol for at least 1 h and subsequently freeze–thawed for three cycles in liquid nitrogen. Cells were then treated with 0.1 N HCl (in 0.7% Triton X-100), incubated in RNase ONE (Promega) for 30 min, dehydrated through a 70–80–100% ethanol series and incubated in 50% formamide, 2 x SSC for at least 2 h prior to hybridization. Probes were hybridized to pretreated slides at 85 °C for 2 min on a heat block and incubated at 37 °C in a humidified chamber overnight. Post-hybridization washes were performed at 42 °C in 2 x SSC/50% formamide followed by washes in 0.1 x SSC at 60 °C. Slides were incubated with Streptavidin-Alexa fluor 555 (Invitrogen) for biotinylated probes or anti-digoxigenin Alexa fluor 488 (Invitrogen) for digoxigenin-labeled probes. After washing, nuclei were counterstained using DAPI and slides mounted with Prolong Gold antifade reagent (Invitrogen). Z-sections were captured using a Nikon 90i eclipse microscope with equivalent exposure times for all images. An average of more than 170 nuclei were analyzed for each probe mix using the Volocity 3D image software (PerkinElmer) to measure center-to-center distances. The individual populations (cyan signals for the interacting regions and magenta signals for the rDNA) were detected separately and only spots within the nucleus (as determined by the DAPI signal) were considered. Furthermore, only cells in which both populations were present in the same nucleus were used for further analysis. By defining the average cell size, individual cells could be discriminated. All distances were measured from the centroids of each population and the minimum distance for each spot selected for statistical analyses, thus accounting for allelic differences.

Significance was assessed using the Wilcoxon–Mann–Whitney test. The BACs used in the experiments were RP23–225M6 (rDNA), RP23–312-I5 (*Ebf1*), RP23–443-E23 (*Chd1l*), RP23–16-E24 (*Parn*), RP23–407-C12 (*Cyp51*), RP23–449-F4 (*Eif3d*), RP23–190-N22 (*Pbx1*), RP23–471-F21 (*Pdp2*), and RP23–448-J8 (*U1*). For the generation of probes, BAC DNA was labeled by nick translation with biotin-16-dUTP or digoxiginin-11-dUTP (Roche) using a nick translation kit (Roche).

### Psoralen cross-linking assay

Psoralen cross-linking^12^ was performed in isolated nuclei that were irradiated in the presence of 10 μg/ml 4,5,8’-trimethylpsoralen (Sigma–Aldrich). Genomic DNA was isolated and 10 μg was digested with SalI (Promega). Fragmented DNA was separated on a 0.9% agarose gel, transferred onto nylon membranes, and immobilized by UV irradiation at 1875 x 100 μJ/cm2 using a UV cross-linker (Stratalinker 2400; Agilent Technologies). The membrane was hybridized with ^32^P-labelled rDNA (generated using a nick translation labeling kit, Roche) and visualized on a PhosphoImager (GE Healthcare). Quantification was performed using ImageQuant (TLv2005.04; GE Healthcare). Original psoralen blot scans that correspond to quantitated and/or edited blot images shown in Figs 1, 2 and Supplementary Fig. 2 are shown in Supplementary Fig. 1a, b and Supplementary Fig. 8. A representative depiction of the psoralen blot quantitation is shown in Supplementary Fig. 1c, d.

### 4C-library preparation

Chromatin libraries for 4C-seq were prepared as previously described^83,84^ and detailed here. Isolated wild type, premalignant and malignant pre-B or harvested Eμ-*Myc* lymphoma cells (LMP and sh*Ubtf*), all performed in two biological replicates, were crosslinked with 1% formaldehyde for 10 min, quenched with 125 mM glycine before being pelleted by centrifugation (320g, 8 min, 4 °C). Cells were suspended in ice cold lysis buffer (10 mM Tris pH 8.0, 10 mM NaCl, 0.2% NP-40, protease inhibitor cocktail-EDTA-free (Roche)) and lysed (10 min). Cell nuclei were collected by centrifugation (600g, 5 min, 4 °C), supernatant removed, and nuclei resuspended in SDS (0.3% final conc., 1 h, 37 °C, with shaking). Triton X-100 (1.8% final conc.) was added to sequester excess SDS and the chromatin solution incubated (1 h, 37 °C, with shaking). Restriction buffer (NEB) was added (1x final conc.) before the chromatin was digested with DpnII (100 U, New England Biolabs, 37 °C, o/n, with shaking). DpnII was inactivated (SDS [1.3% final conc.], 20 min, 65 °C) and the chromatin solution diluted (10-fold) in water. Triton X-100 (1% final conc.) was added and the digested chromatin incubated (1 h, 37 °C, with shaking) to sequester the unreacted SDS. As a control for possible inter-molecular ligation events, an external DNA fragment from *Escherichia coli* (E.coli K12 MG1655, sequence: 29555–29765) was added, at the same concentration as a single copy locus, to each reaction. Fragments were ligated using T4 ligase (20 U; Invitrogen) for 5h at 16 °C before being moved to room temperature for 30 min Cross-links were reversed by the addition of proteinase K (300μg) and incubation at 65 °C (overnight) before treatment with RNase A (300μg, incubation at 37 °C for 1h). Ligation products were purified by phenol-chloroform extraction and ethanol precipitation. For 4C-seq the DNA fragments were amplified by PCR using inverse primers in the rDNA loci (Supplementary Table 1) in the presence of two PCR blockers (Supplementary Table 2), which have a C3 spacer at the 5’ and 3’end, located on either side of the specific restriction fragments in the rDNA^84^. The PCR blockers inhibit elongation and thus amplification of rDNA sequences^85^. By using PCR blockers, amplification of adjacent fragments, caused by incomplete digestion or re-ligation of bait and adjacent regions, will be avoided (see schematic, Supplementary Fig. 6). All primer sequences are listed in Supplementary Tables 1 (bait primers) and 2 (blockers). PCR products were mixed in equimolar ratios and sequenced on an Illumina HiSeq2500 platform at Peter MacCallum Cancer Centre (150bp, paired-end).

### 4C-seq analysis

The sequencing reads were demultiplexed by splitting the reads according to the 5’ bait sequence followed by computational removal of undigested (or digested then re-ligated, both of which are minimized by PCR blockers, Supplementary Fig. 6) or self-ligated reads. Bait sequences were trimmed using cutadapt and the trimmed reads were aligned to the mm9 genome assembly (ENSEMBL *Mus musculus* NCBIM37.67) using bowtie2^86^ with default parameters. Reads for which the bait sequences (up to the DpnII site) were not an exact match were removed and thus interactions originating from 18S and 28S pseudogenes were not considered for downstream analysis. Aligned reads were divided into non-overlapping 5000-bp windows and the average read number calculated as semi-quantitative measure of interaction frequency. To determine the expected interaction frequency relative to TSS a simulated dataset was generated by in silico DpnII digestion, and analysed using the same parameters as for the real dataset (Supplementary Fig. 7). The average distance to TSS was then calculated and compared to the distance measured in wild type, premalignant, and malignant cells. While we observed a similar overall distribution of interacting regions compared with our dataset, our findings deviated in that we saw a greater enrichment of regions around approximately 2kb upstream of the closest TSS, but less so immediately at the TSS (Supplementary Fig. 7), corroborating the non-random nature of our interactions.

To focus on rDNA interactions independent of bait location within the rDNA repeat, we combined the reads for all bait sequences and removed interactions that only appeared in less than four of the 11 bait sequences, and then the average read number was calculated as semi-quantitative measure of interaction frequency. Subsequently, the counts were normalized to the effective library size, which takes the median of the ratio of the count for each gene to the geometric mean of the counts as the scaling factor for each library (see Supplementary Data 1, raw and normalized to effective library size provided). After removing a small number of interactions that displayed very high interaction frequency in only one of the rDNA bait sequences, all normalized interactions were mapped to each chromosome to examine their distribution (Supplementary Figure 3a and b).

Differential interactions were determined using the normalized read count data across replicates and applying a FDR cutoff (FDR ≤ 0.1). The R package DEseq was used for differential interaction analysis. DEseq is considered more stringent than EdgeR, calculating a scaling factor based on mean ratio, which has been shown to perform better in regards to false-positives^87^. Non-differential interactions (no significant change between wild type, premalignant, and malignant; FDR > 0.1), i.e., constitutively associated with the rDNA at all stages, were also identified (Supplementary Data 2). We then focused on differential interactions between premalignant and malignant cells (coincident with rDNA class switching, Supplementary Data 4). Only interactions also identified in Eμ-*Myc*-LMP/Eμ-*Myc*-sh*Ubtf* cells (Supplementary Data 5) were used for further analysis, allowing us to identify and examine UBTF-dependent interactions. We categorized interactions identified in the Eµ-*Myc* pre-B cells into those that increase upon transition from premalignant to malignant and decrease in the absence of UBTF, and interactions that decrease upon transition from premalignant to malignant and increase in the absence of UBTF (Fig. 4a and Supplementary Data 6).

Interacting regions were visualized using circos plots^88^, which were generated using the most significantly differential interactions, where interactions identified in both replicates with one representative bait in the 28S rDNA (read counts > 10) were selected and those with the highest interaction frequency, i.e., the top 10% of interactions (232 in wild type, 493 in premalignant and 1546 in malignant), were used to generate the circos plots. Comparisons of 4C-seq data and RNA-seq data for associated regions were performed by displaying the RPKM distribution of genes interacting and genes not interacting with rDNA as a kernel density estimate. To compare differentially interacting regions with differentially expressed genes, all differentially expressed genes between malignant vs. wild type and malignant vs. premalignant (FDR ≤ 0.1, −0.5 ≥ logFC ≥ 0.5) were grouped into genes associated with increased interacting regions and genes associated with decreased interacting regions and the kernel density of fold change (log2) in expression estimated.

The SPRITE method interrogates crosslinked complexes of DNA segments in physical proximity by marking all reads in a cluster (nuclear components that had been crosslinked together) with the same barcode^62^. SPRITE reads were mapped (mapq > 10) to NCBIM37.67 using Bowtie2 (v2.3.1) with the default parameters (GEO: GSE114242). To find the concordance to our 4C data, we selected all clusters that included rDNA regions 18S and 28S and merged overlapping bins/regions. We then compared the 4C interactions (5-kb bins, merged baits, and replicates) to the regions containing SPRITE interactions. Using the R package ChIPseeker (v1.8.6)^89^, we searched 100-kb up- and downstream of the SPRITE peak (getTagMatrix with windows=SPRITE +/− 100 kb) and plotted the average profile (plotAvgProf, 200-kb region) for the Eµ-*Myc* lymphoma cell lines (LMP, sh*Ubtf*). For the bootstrap control analyses, SPRITE regions were replaced with the same number (and size) of random regions from across the genome. The 4C regions were then mapped to these random regions with the same parameters as with the SPRITE-identified interactions (window size +/− 100 kb around the random bootstrapped regions).

GO analysis was performed using ConsensusPathDB^90–92^ for the large set (9382 unique genes) of constitutive (between wild type, premalignant and malignant) interacting regions and DAVID^93,94^ for the UBTF-dependent differential (between premalignant and malignant) interacting regions. All statistical analyses were performed using the R package (R team-Foundation for Statistical Computing 2010).

### 3C-qPCR

3C libraries were prepared as described for 4C-seq but ending with the extraction of DNA fragments. Quantification of ligated products was performed by Taqman qPCR^95^. The primers used for 3C sample amplification are listed in Supplementary Tables 3 and 4. Primer efficiency and ligation efficiency were determined using randomly ligated BAC DNA containing genomic regions of interest (BACs listed in 3D DNA-FISH methods), recommended as a control for more reliable quantification in the context of complex mouse/human genomes^96^. Further, we employed standard protocols for the generation of our control BAC library^95^. Cross-linking frequencies were normalized to a single copy loci (*Ercc3* gene) and multi copy loci (primer in 28S rDNA) (relative interaction frequencies were determined by qPCR compared to standard curves as previously determined). As internal control, the crosslinking frequency of fragments separated by 2 kb at the *Ercc3* gene was analysed^97^. Results were expressed as interaction frequency in malignant cells relative to premalignant cells.

### FACS analysis and sorting

The following antibodies were used for flow cytometric analysis of cells isolated from bone marrow: APC-conjugated anti-B220/CD-45R (eBioscience), fluorescein isothiocynate (FITC)-conjugated anti-mouse IgM, and PE-conjugated anti-mouse IgD (BD Pharmingen). Stained cells were resuspended in buffer containing 2 μmol/L FluoroGold (hydroxystilbamidine, Molecular Probes, Invitrogen) before sorting on a FACSAria II flow cytometer (BD Biocsiences, San Jose, USA).

### RNA-FISH

Cells were fixed with 2% paraformaldehyde and cytospun on glass slides. Slides were washed twice in 10% deionized formamide/2 x SSC, then hybridized for 3 h at 37 °C in wash buffer containing 10% dextran sulphate, 50 μg/ml BSA, 500 ng/ml tRNA, and 10 μg/ml Cy3-conjugated RNA probe (Sigma) complementary to part of the rapidly degraded ITS region (thus capturing rDNA transcription rate) of the ribosomal DNA. The slides were then washed twice in wash buffer, then three times in 0.1xSSC at 32 °C, before rinsing in PBS and mounting in VectaShield containing DAPI.

### rDNA methylation analyses

HpaII digestion and/or ChIP-CHOP assays^12^ were performed by digesting genomic DNA or DNA isolated following UBTF ChIP with HpaII before qRT-PCR. The relative amount of DNA resistant to HpaII digestion was calculated after normalization to mock-digested DNA. The MeDIP assay was performed as described^12^ by incubating 4 μg of sonicated single stranded (i.e., heat-denatured) genomic DNA with 4 μg anti-5mC antibody for 2 h at 4 °C in 0.14 M NaCl, 16.7 mM Tris, pH 8.0, and 0.05% Triton X-100. DNA-antibody complexes were isolated with protein A-Sepharose beads (Millipore)/salmon sperm DNA for 2 h at 4 °C and following three washes, the DNA was eluted in 50 mM Tris, pH 8.0, 10 mM EDTA, and 0.5% SDS, then purified (including proteinase K digestion) and analyzed by qRT-PCR. The percentage of bound DNA was calculated (subtracting rabbit serum control) after normalization to 20 ng of input DNA. rDNA amplicon primer sequences are listed in Supplementary Table 5.

### RNA extraction and quantitative real-time PCR

RNA was isolated from sorted pre-B cells using the NucleoSpin RNA extraction kit (MACHEREY-NAGEL). cDNA was prepared and qRT-PCR was performed and normalized to *B2M* mRNA levels^44^. qPCR analysis of rDNA transcription rates by assaying amplicons to rapidly processed regions of the pre-rRNA was previously validated in Eμ-*Myc* cells by comparison to [^32^P] orthophosphate pulse labeling followed by northern analysis, i.e., direct measurement via metabolic labeling of the newly synthesized rRNA^37^. Primer sequences are listed in Supplementary Table 5.

### ChIP and ChIP-seq

Standard UBTF ChIP was carried out as previously described^12,13^. The polyclonal rabbit antiserum to UBTF1/2 was generated in-house and ChIP amplicon primer sequences are listed in Supplementary Table 5. ChIP-sequencing of UBTF-immunoprecipitated and input genomic DNA was performed as previously reported^65^. In brief, libraries from two biological replicate experiments (wild type, premalignant, and malignant cells) were prepared and sequenced using the Illumina HiSeq2500 platform at Peter MacCallum Cancer Centre. The 50-bp paired-end sequencing reads were mapped to the mouse genome database mm9 containing one copy of the rDNA (GenBank: BK000964.1) using bowtie2^86^ and duplicate reads were removed using the picard tool MarkDuplicates. Reads were visualized using the Integrative Genomics Viewer (IGV)^98^. Peaks were called by the R package SPP^99^ and then used to perform an Irreproducible Discovery Rate (IDR) analysis^100^, which measures the consistency between ChIP-seq replicates. Common peaks that passed the IDR reproducibility threshold of <0.05 were annotated to the mouse genome (mm9) and used for further intersection/comparison analyses. All analyses were performed using the default options.

### RNA-seq

Sequencing libraries were prepared from two biological replicate experiments (wild type, premalignant, and malignant cells) using the TruSeq RNA sample preparation kit (Illumina) and sequenced on an Illumina HiSeq2500 platform at Peter MacCallum Cancer Centre (50 bp, SE). The generated 50-bp reads were aligned to the genome using TopHat v2.0.8b with default parameters and the reads counted using HTSeq^101^. The differential expression between wild type vs. malignant and premalignant vs. malignant was then calculated utilizing the DEseq package^102^ in R (version 3.0.2) (R team-Foundation for Statistical Computing 2010) and RPKM values calculated (see Supplementary Data 3). Only differentially expressed genes with FDR ≤ 0.1 and −0.5 ≥ logFC ≥ 0.5 were considered for further analysis.

### Immunofluorescence

Immunofluorescence followed by rDNA FISH was performed as previously described^37,65^. Briefly, cells were fixed in a 2% paraformaldehyde suspension, spun onto slides and stained with a fibrillarin (FLB) antibody (Abcam, ab5821) followed by the 594-conjugated goat anti-rabbit IgG secondary (Molecular Probe, A21442). Cells were fixed again with 4% paraformaldehyde and subjected to rDNA FISH (detailed in Methods, 3D DNA-FISH) and then visualized on an Olympus BX-51 microscope, with images captured using the SPOT Advance image acquisition software (Diagnostic Instruments).

### Immunoblotting

Western blot analysis was performed with equal total protein from whole cell lysate samples resolved by SDS-PAGE and transferred onto PVDF membranes (Millipore). The in-house polyclonal rabbit antisera anti-UBTF1/2 antibody (also used for ChIP-seq) was detected following incubation with anti-rabbit secondary antibody conjugated with horseradish peroxide (HRP) with enhanced chemiluminescence (ECL) reagent (GE Healthcare).

## Supplementary Information

Supplementary Information

Supplementary Data 1

Supplementary Data 2

Supplementary Data 3

Supplementary Data 4

Supplementary Data 5

Supplementary Data 6

Supplementary Data 7

Supplementary Data 8

Description of Additional Supplementary Files

## Data Availability

All data (4C-seq, RNA-seq and UBTF ChIP-seq) have been deposited in the NCBI Gene Expression Omnibus (GEO, http://www.ncbi.nlm.nih.gov/geo/) and are available under accession number GSE70226.
