# Supplementary material for: Changes in long-range rDNA-genomic interactions associate with altered RNA polymerase II gene programs during malignant transformation
Source: Commun Biol. 2019 Jan 28;2:39. doi: 10.1038/s42003-019-0284-y (PMC6349880; doi:10.1038/s42003-019-0284-y)
Supplement: Supplementary file 1 — Supplementary Information [file 42003_2019_284_MOESM1_ESM.pdf]

Supplementary Figure 1

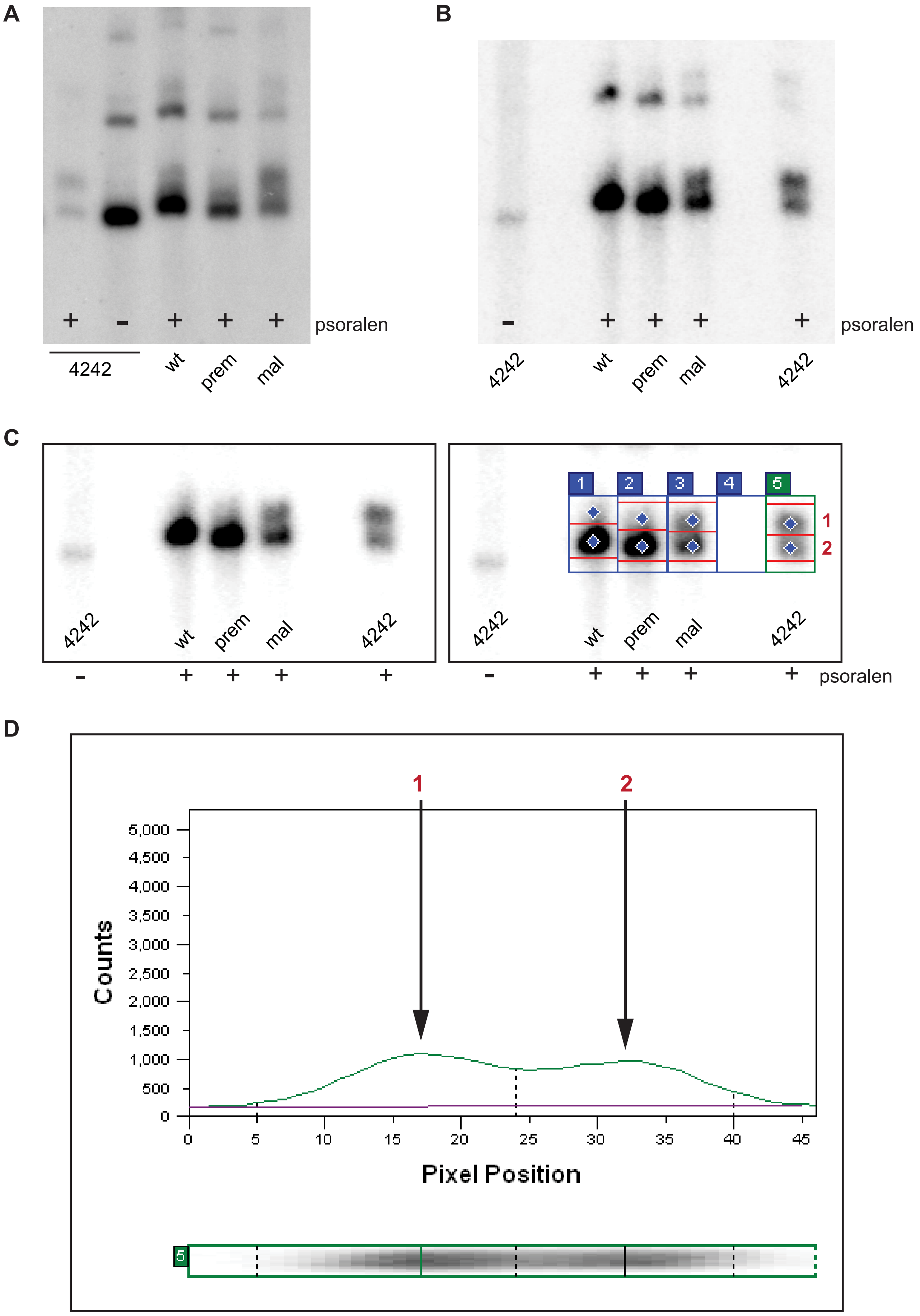

Supplementary Figure 1: Quantitation of active and inactive fractions of psoralen cross-linked rDNA chromatin. (**A-B**) PhosphorImage (Typhoon FLA 7000, GE) scans from 2 representative biological replicate Southern blots of psoralen cross-linked genomic rDNA from wt, prem and mal Eμ-*Myc* cells and the established Eμ-*Myc* 4242 lymphoma line cross-linked both in the absence (-) and presence (+) of psoralen as indicated. (**C**) Representative blot shown without (top) and with (bottom) regions selected for quantitation by ImageQuant TL software. (**D**) Image file from ImageQuant TL of histograms showing pixel counts (y-axis) and relative pixel position (x-axis). Maximum peaks were identified (denoted by blue diamonds, (C) bottom panel) and pixel regions for quantification are numbered (red numbers 1, 2, (C) bottom panel, (D)).

Supplementary Figure 2

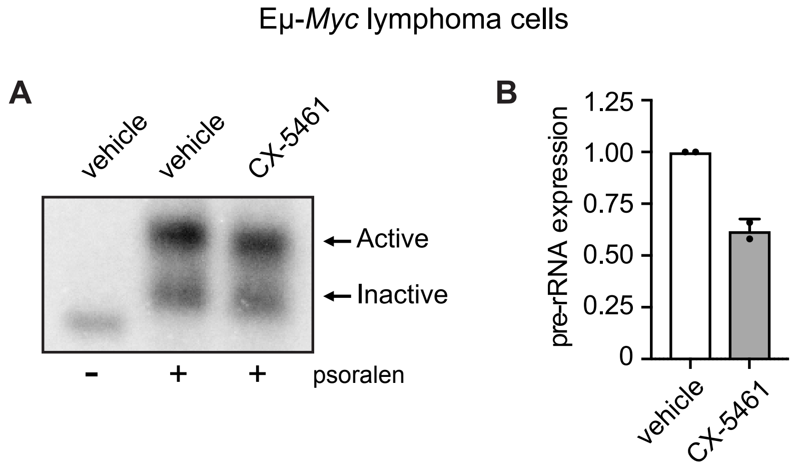

Supplementary Figure 2: rDNA class switching is not impacted by inhibition of Pol I transcription. Eμ-*Myc* lymphoma cells were treated with the selective Pol I transcription inhibitor (50 nM CX-5461) as compared to vehicle (NaH2PO4) for 1 hr. (**A**) Representative Southern blot of psoralen cross-linked genomic rDNA as in Fig.1C, arrows indicate active and inactive rDNA repeats. (**B**) Relative pre-rRNA (47S rRNA) expression determined by qRT-PCR. Pre-rRNA levels were determined as in Fig.1A. RNA levels were normalized to *B2M* mRNA and are represented relative to vehicle (mean ± s.d.; n=2).

Supplementary Figure 3

A

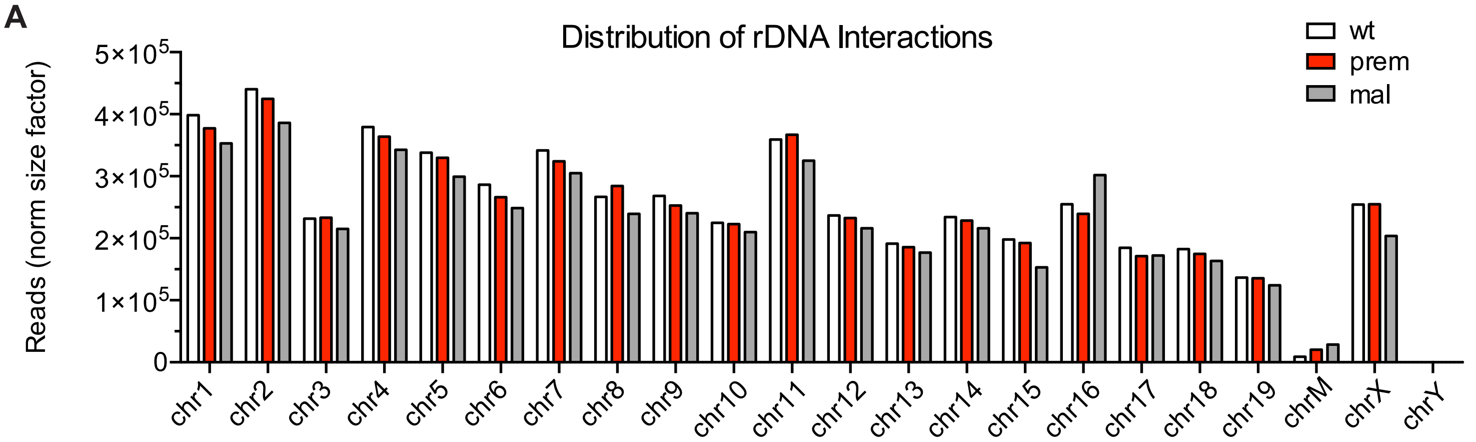

B

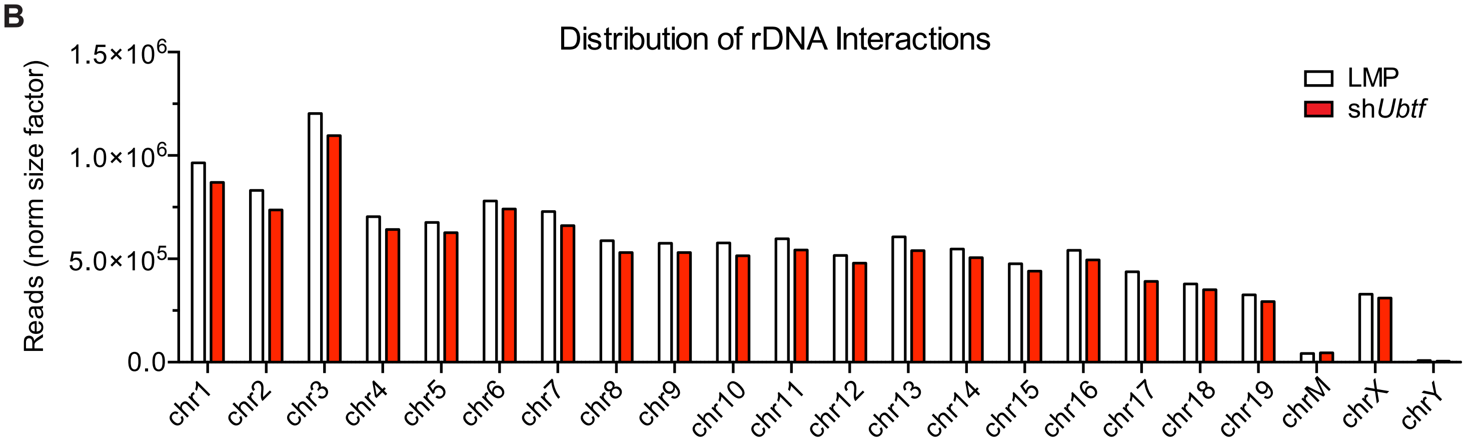

C

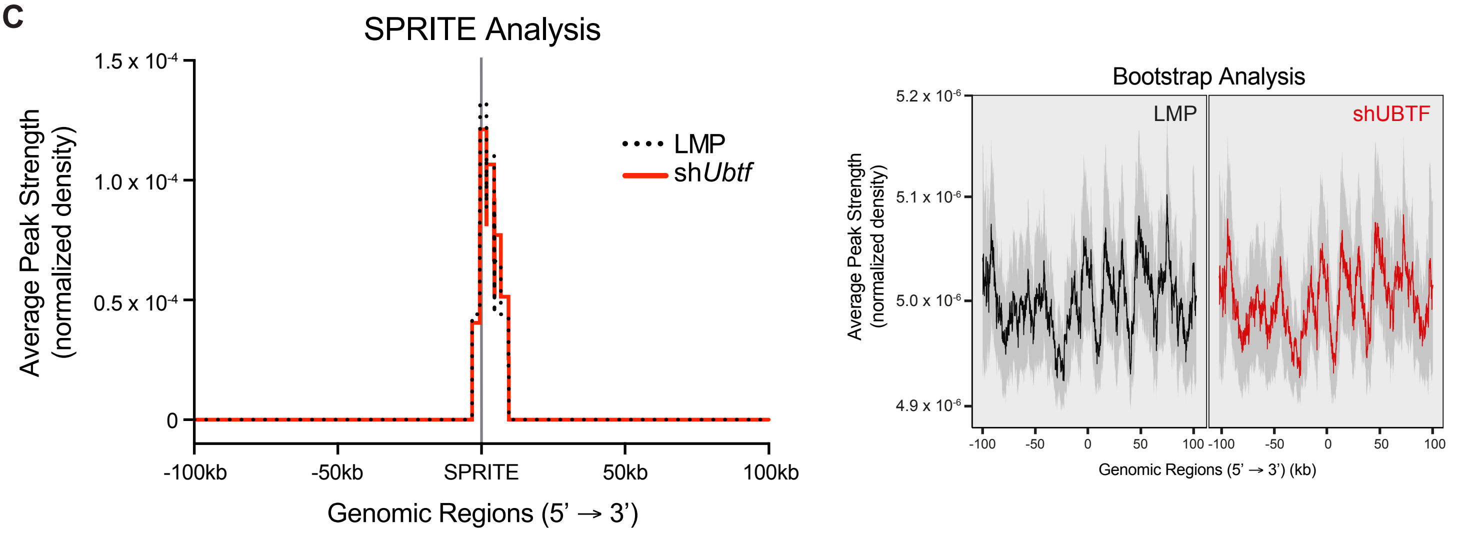

Supplementary Figure 3: Qualitative analyses and validation of 4C-seq data. A uniform distribution of interacting regions was observed among chromosomes in wt, prem and mal Eμ-*Myc* cells and Eμ-*Myc*-LMP compared to Eμ-*Myc*-sh*Ubtf* cells. The distribution of interactions was determined to survey whether the chromosomes containing rDNA arrays (12, 15, 18 and 19) showed a higher fraction of mapped reads compared to the remaining chromosomes due to added intra-chromosomal interactions. After removing interactions that only appeared in less than 4 of the 11 bait sequences or interactions that displayed very high interaction frequency in only one of the rDNA bait sequences, interactions (normalized to effective library size) were mapped to each chromosome for (**A**) wt, prem and mal cells (all read counts listed in Supplementary Data 1) and for (**B**) UBTF knockdown (sh*Ubtf*) and control (LMP) cells (all read counts listed in Supplementary Data 5). (**C**) ChIPseeker tag matrix analyses of the concurrence between 4C rDNA-NAD and SPRITE-identified[1] genomic connections within the 18S and 28S rDNA clusters. Average peak strength (normalized density) of all 4C-seq rDNA interactions (y-axis) in the regions 100kb up- and down-stream of the SPRITE genomic regions (x-axis, peak center denoted by gray vertical line) have a strong peak concordance for both the LMP (black) and shUbtf (red) conditions (left panel). To control for potential random concurrence, a bootstrap analysis shows that 4C-identified interactions mapped to random regions using the same parameters as with SPRITE-identified interactions randomly fluctuate with no correlation of peaks to the plot center. The 10,000 bootstraps are represented as mean (solid line) and 95% confidence interval (dark gray region).

Supplementary Figure 4

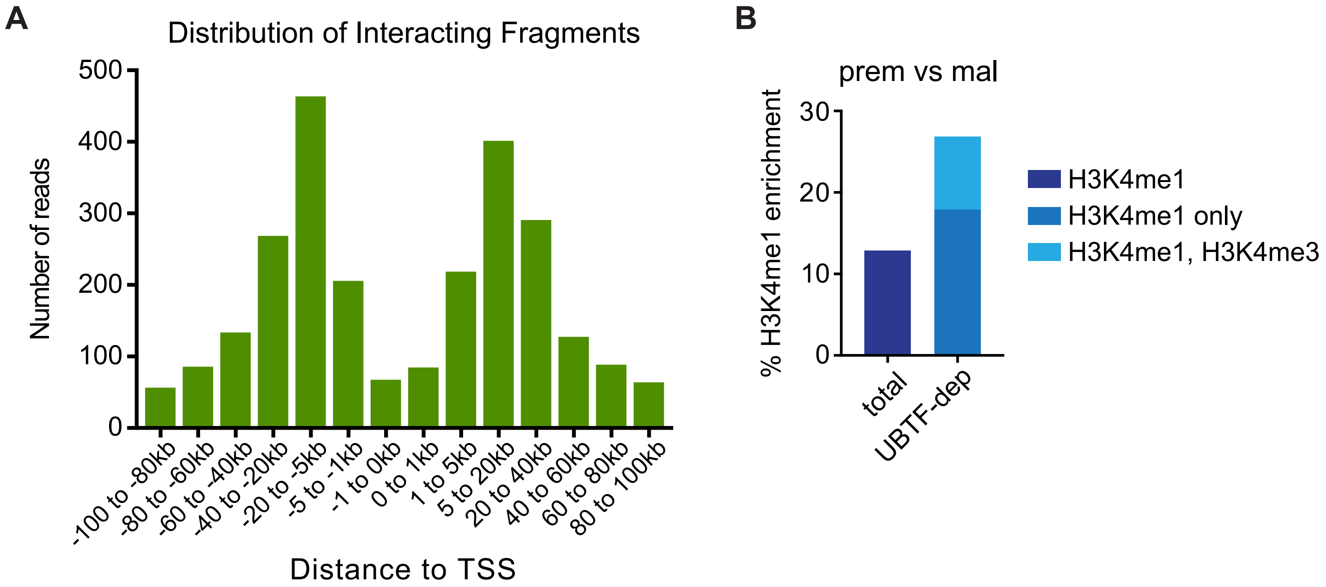

Supplementary Figure 4: UBTF-dependent rDNA-NAD interactions are enriched for histone modifications associated with enhancers and transcriptionally poised genes. (**A**) Distance of rDNA class switch interacting fragments within 100 kb relative to TSS of the nearest gene. (**B**) Enrichment of the gene enhancer H3K4me1 histone modification in total and UBTF-dependent differential rDNA-genome interacting regions based on percentage (%) of overlap of H3K4me1 sites using ChIP-seq previously performed in Eμ-*Myc* tumor cells[2]. Further overlap with sites from ChIP-seq[2] of H3K4me3 histone modifications marking active genes demonstrates the fraction of UBTF-dependent rDNA interacting regions co-enriched for H3K4me1 and H3K4me3 or H3K4me1only.

Supplementary Figure 5

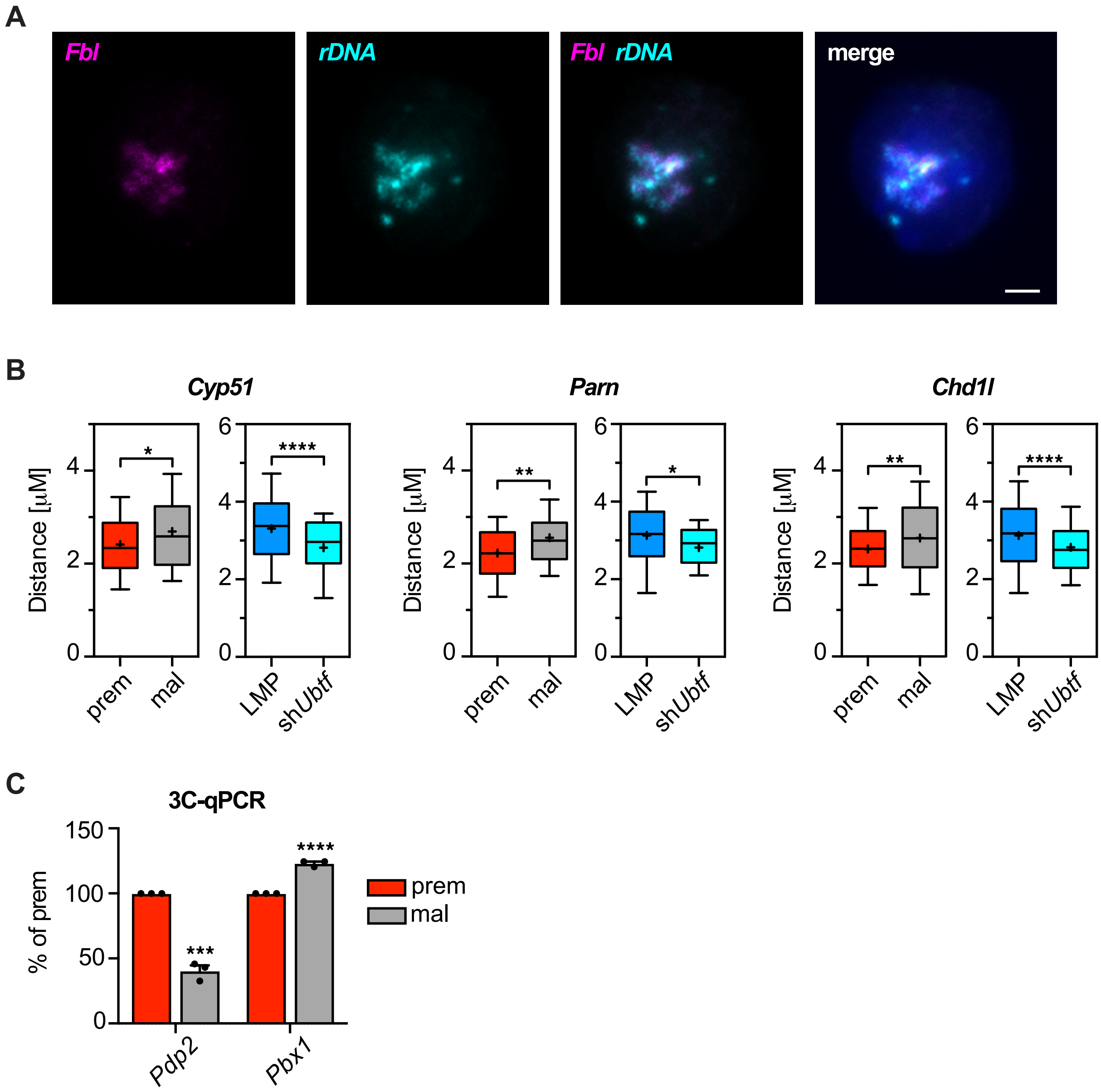

Supplementary Figure 5: Validation of altered rDNA interactions with candidate genomic loci. (**A**) Verification of rDNA FISH (cyan, Alexa 488 signal) staining specificity by overlap with fibrillarin (FBL) immunofluorescence (magenta, Alexa 594 signal), both with (merge) and without DAPI counterstain (blue) in Eµ-*Myc* lymphoma cells. Scale bar, 2.5 µm. (**B**) As described in Fig.7B, boxplots of distances measured from 3D-FISH analysis between rDNA and the following genes showing decreased interactions (distances increasing) from prem to mal that are reversed upon Ubtf knockdown: *Cyp51*, *Parn*, and *Chd1l*. Significance was assessed using the Wilcoxon–Mann–Whitney test (*p-value < 0.05, **p-value < 0.01, ****p-value < 0.0001, n > average 170 distances). (**C**) 3C-qPCR validation of interaction frequency between rDNA and *Pdp2* (decreasing) and *Pbx1* (increasing) from prem to mal cells as described in Fig. 5C (mean ± s.e.m., ***p-value < 0.001; ****p-value < 0.0001; n=3).

Supplementary Figure 6

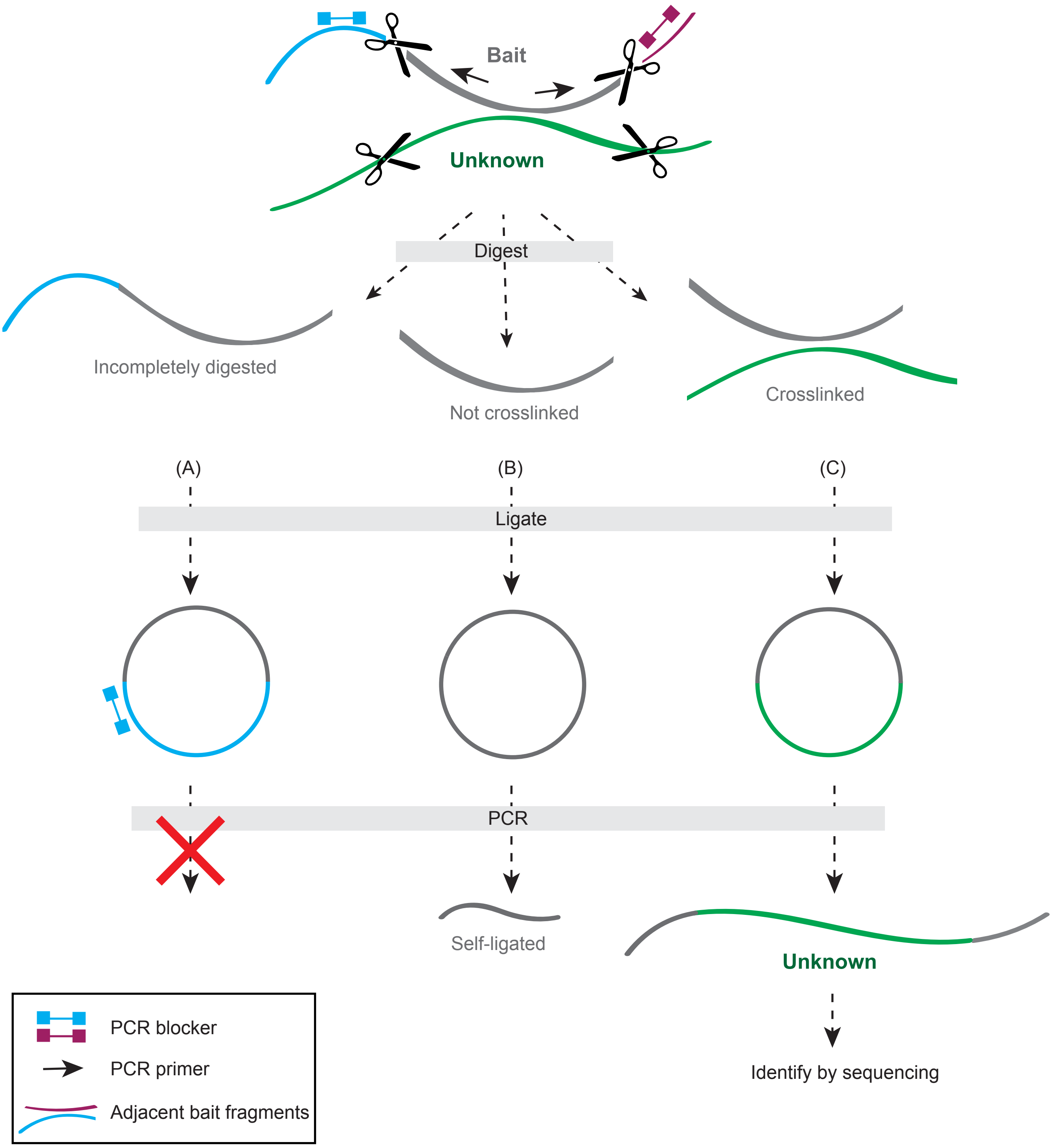

Supplementary Figure 6: Role of C3 PCR blockers in the 4C protocol. Ligation of cross-linked complexes following digestion potentially results in multiple products. (**A**) Incomplete digestion or re-ligation of the digested bait adjacent to the restriction site results in the circularization of the bait and adjacent fragment. The inclusion of a PCR blocker that is homologous to the adjacent region blocks the polymerase and thus prevents PCR amplification from the bait. Thus, there is reduced amplification of the immediately adjacent fragments. (**B**) Circularization of the bait fragment alone results in the production of short PCR products. These products are removed by column purification. (**C**) Circularization of the bait and an unknown restriction fragment leads to the production of chimeric amplicons consisting of the bait sequences and the unknown restriction fragments, which are subsequently identified by sequencing.

Supplementary Figure 7

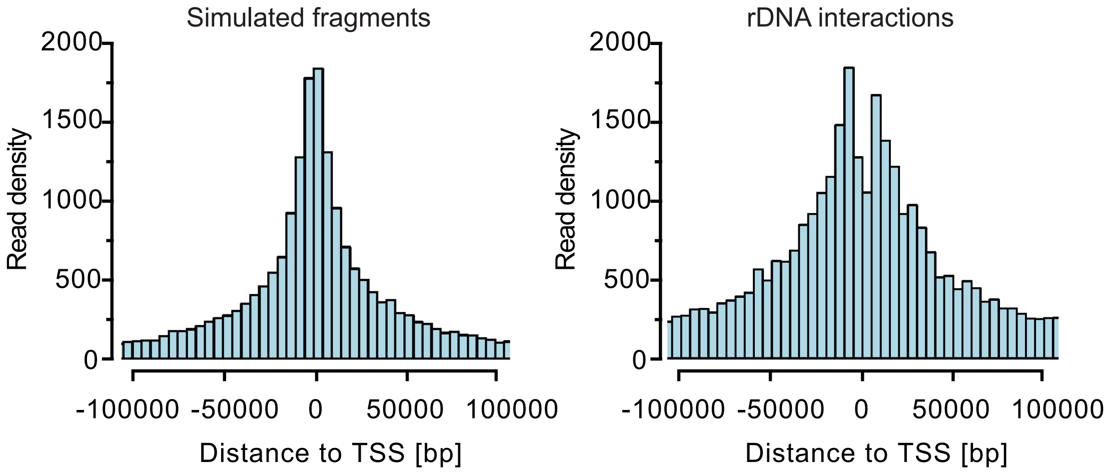

Supplementary Figure 7: Non-random distribution of interacting regions around the TSS. Interaction frequency relative to TSS was determined in a simulated dataset generated by *in silico* DpnII digestion (left panel) and from the actual prem cell 4C data (right panel).

Supplementary Figure 8

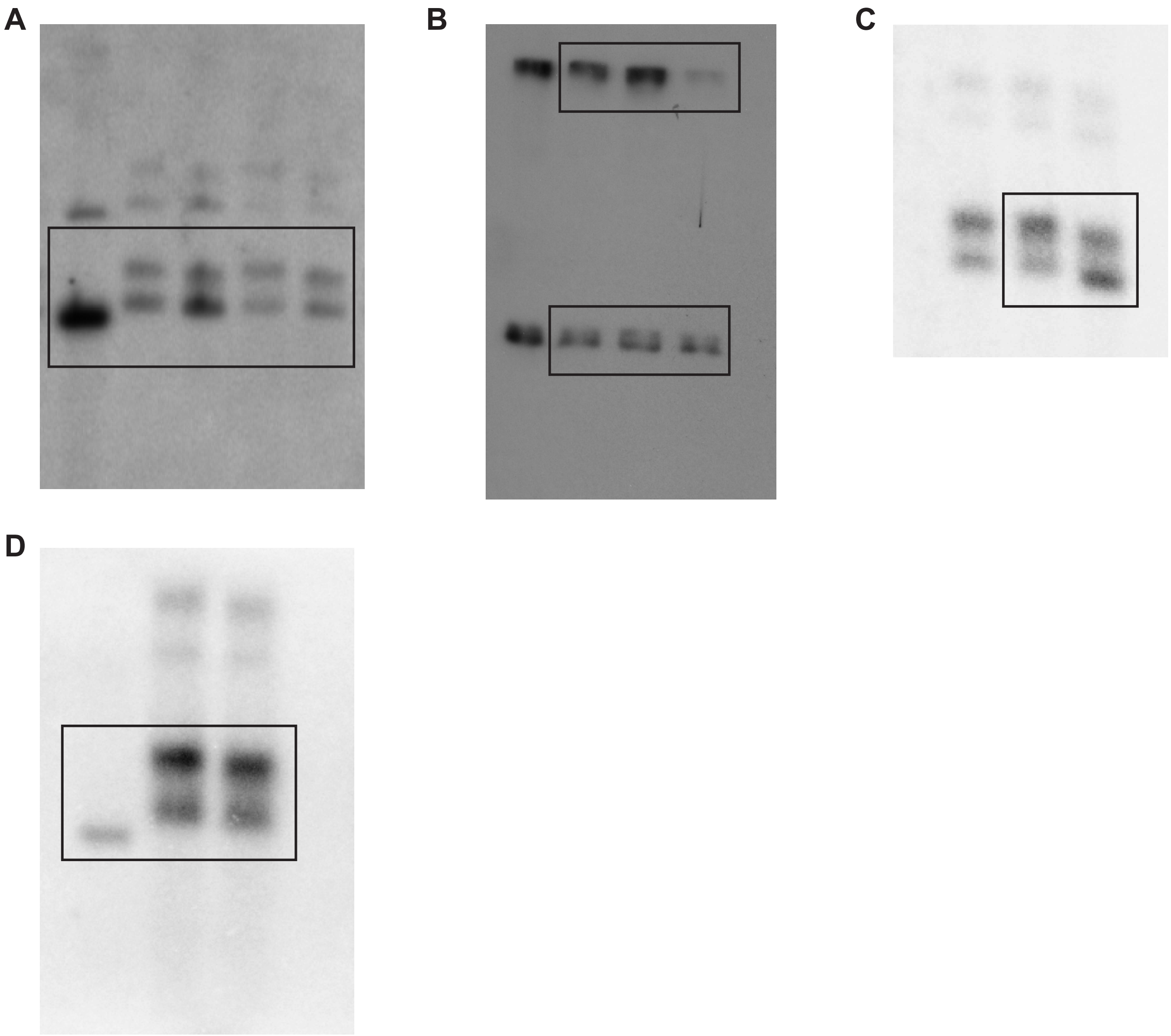

Supplementary Figure 8: Unedited psoralen and western blots shown in the main figures. Black boxes correlate with the edited images shown in the main figures. (**A**) Original psoralen blot shown in Figure 1E (original psoralen blot shown in Figure 1C is presented in Supplementary Figure 1A). (**B**) Original western blot shown in Figure 2A and (**C**) original psoralen blot shown in Figure 2B. (**D**) Original psoralen blot shown in Supplementary Figure 2.

Supplementary Table 1: 4C-seq primer sequences

**Target**	**Sequence**
4020_rDNA	5'-CTAGAGCTAATACATGCCGACG-3'
4020_rDNA	5'-AATCTTTGAGACAAGCATATG-3'
4237_rDNA	5'-GCGGCTTTGGTGACTCTAGA-3'
4237_rDNA	5'-GAGGGAGCTCACCGGGTT-3'
4323_rDNA	5'-GGCCCTGTAATTGGAATGAGT-3'
4323_rDNA	5'-GCAGACGTTCGAATGGGTC-3'
5065_rDNA	5'-GTAACCCGTTGAACCCCATT-3'
5065_rDNA	5'-TCGTTTATGGTCGGAACTACG-3'
5617_rDNA	5'-CGCTACTACCGATTGGATGG-3'
5617_rDNA	5'-TCCTCGTTCATGGGGAATAA-3'
5755_rDNA	5'-GTAGGTGAACCTGCGGAAG-3'
5755_rDNA	5'-CAAGTTCGACCGTCTTCTCA-3'
8343_rDNA	5'-CGCCCGGAGGATTCAAC-3'
8343_rDNA	5'-GCTACCGGCCTCACACC-3'
9375_rDNA	5'-CCCGACGTACGCAGTTTTAT-3'
9375_rDNA	5'-CTCGGCGGACTGGAGAGG-3'
9712_rDNA	5'-CTGCGGTGAGCCTTGAAGC-3'
9712_rDNA	5'-CCCATTTAAAGTTTGAGAATAGGTTG-3'
10426_rDNA	5'- AGGTAAGGGAAGTCGGCAAG-3'
10426_rDNA	5'- CCTGCCCTTCACAAAGAAAA-3'
12134_rDNA	5'- CGTAGACGACCTGCTTCTGG-3'
12134_rDNA	5'- TAGGAAGAGCCGACATCGAA-3'

Supplementary Table 2: 4C-seq blocker sequences

4020-1_blocker	/5SpC3/AACCCGGTGAGCTCCCTC/3SpC3/
4020-2_blocker	/5SpC3/TTCGCTCGCGCTTCCTTA/3SpC3/
4237-1_blocker	/5SpC3/CAGTGAAACTGCGAATGGCT/3SpC3/
4237-2_blocker	/5SpC3/TCGAACGTCTGCCCTATCAA/3SpC3/
4323-1_blocker	/5SpC3/ATCCATTGGAGGGCAAGTCT/3SpC3/
4323-2_blocker	/5SpC3/TCGAACGTCTGCCCTATCAA/3SpC3/
5065-1_blocker	/5SpC3/CCCAGTAAGTGCGGGTCATA/3SpC3/
5065-2_blocker	/5SpC3/TCGGAACTGAGGCCATGATT/3SpC3/
5617-1_blocker	/5SpC3/CTACACTGACTGGCTCAGCG/3SpC3/
5617-2_blocker	/5SpC3/GCTGAGAAGACGGTCGAACT/3SpC3/
5755-1_blocker	/5SpC3/TCGGAACTGAGGCCATGATT/3SpC3/
5755-2_blocker	/5SpC3/CGTGTGGAGCGAGGTGTCT/3SpC3/
7048-1_blocker	/5SpC3/CTACGCCTGTCTGAGCGTC/3SpC3/
7048-2_blocker	/5SpC3/CTCCTCGCTCTCTTCTTCCC/3SpC3/
8343-1_blocker	/5SpC3/GTCGTTCCCCTCTTCCTCC/3SpC3/
8343-2_blocker	/5SpC3/GAGTGAACAGGGAAGAGCCC/3SpC3/
9375-1_blocker	/5SpC3/GTCGTTCCCCTCTTCCTCC/3SpC3/
9375-2_blocker	/5SpC3/GGCCACTTTTGGTAAGCAGA/3SpC3/
9712-1_blocker	/5SpC3/ATGGTGAACTATGCTTGGGC/3SpC3/
9712-2_blocker	/5SpC3/AAGGGTTCCATGTGAACAGC/3SpC3/
10426-1_blocker	/5SpC3/ACTTCGGGATAAGGATTGGC/3SpC3/
10426-2_blocker	/5SpC3/GGCGTCCAGTGCGGTAAC/3SpC3/
12134-1_blocker	/5SpC3/CAGGGATAACTGGCTTGTGG/3SpC3/
12134-2_blocker	/5SpC3/AAGTCAGCCCTCGACACAAG/3SpC3/

Supplementary Table 3: 3C-qPCR primer sequences

Target	5'primer	3'primer
rDNA-47s	CCCGACGTACGCAGTTTTATCCGGTAAAGC GAATGATTAGAGGTCTTGGGGCCGAAAC	
Ebf1	AGTAACACTCTCTACACCATGGG	
Pdp2	CTCCAGCAAGCAAGAGGGG	
Pbx1	TGTTCTTGGATTCGGTTAGCGA	
rDNA-28s	GCGTTAGGACCCGAAAGATG	GAGGGAAACTTCGGAGGGAA
Ercc3	TCTTTCCTTTCCTTTCCTTTCC	CTCTTTCTGGGAGTTTTCATGT

Supplementary Table 4: 3C-qPCR taqman probes

Taqman probes	Sequence
rDNA-47s probe	/56-FAM/AGAGGTCTT/ZEN/GGGGCCGAAAC/3IABkFQ/
rDNA-18s probe	/56-FAM/TTTGGTCGC/ZEN/TCGCTCCTCTC/3IABkFQ/
rDNA-28s probe	/56-FAM/CCGACCTGG/ZEN/GTATAGGGGCG/3IABkFQ/
Ercc3 probe	/56-FAM/CCTTCATCA/ZEN/GTGATGCGCTCC/3IABkFQ/

Supplementary Table 5: qRT-PCR and ChIP amplicon primer sequences

Target	5'primer	3'primer
47S-5'ETS	ACACGCTGTCCTTTCCCTATTAACA CTAAA	AGTAAAAAGAATAGGCTGGACAAGC AAAAC
Ubtf1/2	CGCGCAGCATACAAAGAATACA	GTTTGGGCCTCGGAGCTT
B2m	TTCACCCCCACTGAGACTGAT	GTCTTGGGCTCGGCCATA
rDNA-ENH	AGGAGGCCGGGCAAGCA	CCTCCTTGTTAGAGACCGTCCTTAA
rDNA-UCE	AGTTGTTCCTTTGAGGTCCGGT	AGGAAAGTGACAGGCCACAGAG
rDNA-CORE	AGTTGTTCCTTTGAGGTCCGGT	CAGCCTTAAATCGAAAGGGTCT
rDNA-ETS1	CCAAGTGTTCATGCCACGTG	CGAGCGACTGCCACAAAAA
rDNA-ITS1	CCGGCTTGCCCGATTT	GCCAGCAGGAACGAAACG
rDNA-28S	AGTAGCAAATATTCAAACGAGAACTTTG	ACCCATGTTCAACTGTTCA
rDNA-28S-2	CTCCCGACGTACGCAGTTTTATCC	ATCGTTTCGGCCCCAAGACC
rDNA-IGS2	ACTTGCAAACCGGGCCACTAAA	TTCCTTGTTCTGTCACTCGGTTGC

Supplementary References

1. Quinodoz, S. A. *et al.* Higher-order inter-chromosomal hubs shape 3D genome organization in the nucleus. *Cell* **174,** 744-757 (2018).
2. Sabò, A. *et al.* Selective transcriptional regulation by Myc in cellular growth control and lymphomagenesis. *Nature* **511,** 488–492 (2014).
