# Supplementary material for: Changes in long-range rDNA-genomic interactions associate with altered RNA polymerase II gene programs during malignant transformation
Source: Commun Biol. 2019 Jan 28;2:39. doi: 10.1038/s42003-019-0284-y (PMC6349880; doi:10.1038/s42003-019-0284-y)
Supplement: Supplementary file 10 — Description of Additional Supplementary Files [file 42003_2019_284_MOESM10_ESM.pdf]

Description of Supplementary Data Excel files

Supplementary Data 1: All rDNA interactions

4C-seq was used to identify long-range chromatin interactions that occur with rDNA sequences in isolated wt, prem and mal cells. 11 bait sequences located in the transcribed 18S and 28S rDNA regions (Fig. 3a) were used to capture interactions between the rDNA and rest of the genome. rDNA interactions were included in subsequent analyses if they were observed in two biological replicates (i.e. animals) per cell stage. We combined all replicated rDNA interactions for each cell type and calculated the average read count per interaction in 5kb non-overlapping windows as a semi-quantitative measure of interaction frequency. Shown are both raw read counts and read counts normalized to the effective library size and the interacting regions are annotated with the nearest associated gene.

Supplementary Data 2: Constitutive rDNA interactions

4C-seq identified interacting regions from Suplementary Data 1 that show no significant change (FDR > 0.1) between any pair of the wt, prem and mal cell stages (constitutive rDNA interactions). Shown are both raw read counts and read counts normalized to the effective library size and the interacting regions are annotated with the nearest associated gene. These data were used to perform the analyses shown in Fig. 3c and Table 1.

Supplementary Data 3: RPKM & FC gene expression

RNA-seq on wt, prem and mal cells determined the average gene expression (reads per kilobase of transcript per million reads mapped [RPKM]) for all genes (highlighted in yellow). From this, we determined the expression fold change (logFC) and then the significant differentially expressed genes (FDR ≤ 0.1; -0.5 ≥ logFC ≥ 0.5) between wt and mal cells (highlighted in green) and prem and mal cells.

Supplementary Data 4: Differential rDNA interactions between prem and mal cells

4C-seq identified interacting regions from Suplementary Data 1 that are significantly changed (differential rDNA interactions, FDR < 0.1) between prem and mal cell stages, coincident with rDNA class switching. Shown are the logFC in interaction and the interacting regions are annotated with the nearest associated gene. These interaction data along with gene expression changes from prem to mal were used to perform the analyses shown in Fig. 3e.

Supplementary Data 5: Differential rDNA interactions between LMP and *Ubtf* KD cells

4C-seq was used to identify long-range chromatin interactions that occur with rDNA sequences in stable *Ubtf* knockdown lymphoma cells (Eμ-*Myc*-sh*Ubtf*) compared to control cells (Eμ-*Myc*-LMP) as described for lymphoma progression cells. Shown are both raw read counts and

read counts normalized to the effective library size and the interacting regions are annotated with the nearest associated gene.

Supplementary Data 6: rDNA class switch differential rDNA interactions

4C-seq identified rDNA interactions detected in the *Ubtf* knockdown cells were intersected with those from the Eμ-*Myc* pre-B cells and reciprocal interactions that either increase upon prem to mal transition and decrease with UBTF loss (1,822 interactions) or decrease upon prem to mal transition and increase with UBTF loss (1,246 interactions) (work flow schematic shown in Figure 4a) were retained for all subsequent analyses. These UBTF-dependent rDNA interactions were termed rDNA class switch interactions. Shown are the logFC in interaction for prem to mal and LMP to shUbtf and the interacting regions are annotated with the nearest associated gene.

Supplementary Data 7: UBTF ChIP-seq IDR identified peaks and associated genes in prem and mal cells

UBTF ChIP-seq was performed in wt, prem and mal cells and peaks called by the R package SPP were used to perform an Irreproducible Discovery Rate (IDR) analysis between ChIP-seq replicates. Common peaks (i.e. identified in both replicate samples, peak regions from both replicates shown) that passed the IDR reproducibility threshold of <0.05 were annotated and used for further intersection/comparison analyses. UBTF-enriched peaks (non-rDNA repeat) from prem and mal cells (shown in the **prem IDR replicate peaks** and **mal IDR replicate peaks** sheets) were intersected with the rDNA class switch interactions identified in Supplementary Data 6 and any regions with an overlap of 1 bp or more were considered overlapping (analyses shown in Fig. 4c). We then intersected the nearest associated gene lists from these same sets and the common genes (prem, 130 genes; mal, 189 genes) are listed in the **prem genes_overlap** and **mal genes_overlap** sheets (analyses shown in Fig. 4c).

Supplementary Data 8: Gene ontology analysis of differentially expressed genes associated with rDNA class switch rDNA interactions

Gene ontology (GO) analysis was performed on genes associated with rDNA class switch interactions that showed either increased rDNA interaction/decreased gene expression or decreased rDNA interaction/increased gene expression (wt to mal) during malignant transformation. Shown here are the expanded GO results using standard DAVID analysis (summarized in Fig. 4d), with the individual genes listed under the enriched GO terms from genes with decreased expression (**UBTF-dependent increased** interactions sheet) and genes with increased expression (**UBTF-dependent decreased** interactions sheet) and the detailed statistical analysis of each enriched term.
